# Tumor acidity activated triphenylphosphonium-based mitochondrial targeting nanocarriers for overcoming drug resistance of cancer therapy

**DOI:** 10.7150/thno.35748

**Published:** 2019-09-21

**Authors:** Hui Yu, Jia-Mi Li, Kai Deng, Wei Zhou, Cai-Xia Wang, Qian Wang, Kun-Heng Li, Hong-Yang Zhao, Shi-Wen Huang

**Affiliations:** Key Laboratory of Biomedical Polymers of Ministry of Education, Department of Chemistry, Wuhan University,Wuhan 430072, China

**Keywords:** tumor acidity, charge reversal, mitochondrial targeting, resistance, cancer therapy

## Abstract

The drug resistance in cancer treatment with DOX is mainly related to the overexpression of drug efflux proteins, residing in the plasma and nuclear membranes. Delivering DOX into the mitochondria, lacking drug efflux proteins, is an interesting method to overcome DOX resistance. To solve the problem of positively charged triphenylphosphonium (TPP) for mitochondrial targeting *in vivo*, a charge reversal strategy was developed.

**Methods:** An acidity triggered cleavable polyanion PEI-DMMA (PD) was coated on the surface of positively charged lipid-polymer hybrid nanoparticle (DOX-PLGA/CPT) to form DOX-PLGA/CPT/PD *via* electrostatic interaction. The mitochondrial localization and anticancer efficacy of DOX-PLGA/CPT/PD was evaluated both *in vitro* and in *vivo*.

**Results:** The surface negative charge of DOX-PLGA/CPT/PD prevents from rapid clearance in the blood and improved the accumulation in tumor tissue through the enhanced permeability and retention (EPR) effect. The hydrolysis of amide bonds in PD in weakly acidic tumor tissue leads to the conversion of DOX-PLGA/CPT/PD to DOX-PLGA/CPT. The positive charge of DOX-PLGA/CPT enhances the interaction with tumor cells, promotes the uptake and improves DOX contents in tumor cells. Once endocytosed by tumor cells, the exposed TPP in nanomedicine results in effective mitochondrial localization of DOX-PLGA/CPT. Afterward, DOX can release from the nanomedicine in the mitochondria, target mtDNA, induce tumor cells apoptosis and overcome DOX resistance of MCF-7/ADR breast cancer.

**Conclusion:** Tumor acidity triggered charge reversal of TPP-containing nanomedicine and activation of mitochondrial targeting is a simple and effective strategy for the delivery of DOX into the mitochondria of cancer cells and overcoming DOX resistance of MCF-7/ADR tumor both *in vitro* and *in vivo*, providing new insight in the design of nanomedicines for cancer chemotherapy.

## Introduction

Despite the severe adverse effects on the patients, chemotherapy is still one of the most important approaches and has been widely used in clinical cancer treatments. Unfortunately, the drug resistance often occurred shortly after chemotherapy in the clinic 1,2. Furthermore, in many cases, the chemotherapeutic treated tumors exhibit multidrug resistance (MDR). MDR is one of the biggest challenges in cancer chemotherapy and has resulted in more than 90% of clinical chemotherapy failures 3,4. The mechanism of cancer cell MDR is complicated and dependent on the kinds of cancers, which has not been completely understood and needs further investigation 5. Multiple MDR mechanisms, including intracellular and extracellular drug resistance mechanisms, have been found 6. It is crucial to develop drug resistance mechanism-guided new formulations to overcome MDR in cancer chemotherapy. The rapid progress in nanomedicine provides a variety of new weapons to fight against cancer MDR 7-9, including stimulus-responsive nanocarriers for the delivery of chemotherapeutic 10-13 and the combinational therapy of chemotherapeutic with small molecule inhibitors 14,15 and/or nucleic acid drugs 16. Most work in the past decades focused on the design of nanocarriers to target cancer cells alone and improve anticancer efficacies by increasing the uptake, retention of nanomedicine in cancer cells and responsive release of chemotherapeutic, or modulating the microenvironment inside cancer cells to synergistically combat MDR cancer. Additionally, subcellular targeting delivery of chemotherapeutic, such as mitochondrial targeting 17 and nucleic targeting 18, is an alternative strategy to enhance anticancer ability and reverse cancer MDR. Some of recent work targeted both cancer cells and modulating tumor microenvironment (TME), such as abnormal tumor vasculature 19, hypoxia 20 and tumor-associated macrophages 21 etc., to reverse MDR in cancer chemotherapy.

Doxorubicin (DOX), the first anthracycline drug, has shown excellent antitumor ability and has been widely used as a frontline drug for the treatment of various cancers, such as leukemia, Hodgkin's lymphoma, bladder and breast cancers, etc. The nucleic DNA is the main target of DOX in cancer cells. DOX can cross cell membrane, karyotheca and localize in the nucleic to intercalate into DNA base pairs, generate topoisomerase II (TOPOII)-mediated nuclear DNA lesion and induce cell apoptosis. However, the outcomes of DOX in clinical cancer therapy are limited due to the severe cardiotoxicity and the development of MDR, which leads to treatment failure. It is common to result in overexpression of drug efflux proteins residing in the plasma and nuclear membranes of cancer cells after treatment with DOX 22,23. The drug efflux proteins in cancer cells, mainly including P-glycoprotein (P-gp), are able to pump the drug out of the cells and decrease the content of DOX, leading to failure in killing cancer cells 24.

The mitochondrion is not only the powerhouse of a cell, but also the center to regulate cell death. Therefore, mitochondrion is always considered as an ideal target for cancer therapy 25-27. Similar to the nucleus, there are nucleic acids in the mitochondria of cells. The previous work demonstrated that mitochondrial targeting DOX derivative maintained the ability to target TOPOII in the mitochondria and damaged mtDNA in DOX-resistant cancer cells, implying the importance of mitochondrion as an alternative target of DOX for MDR cancer therapy 22. Due to free DOX is difficult to cross highly impermeable mitochondrial membrane, many efforts have been made for targeting delivery of DOX into the mitochondria of cancer cells, mainly including mitochondrial targeting ligand-conjugated DOX and DOX-loaded mitochondrial targeting nanomedicines 28-31. The mitochondria are highly negatively charged (-160 mV to -180 mV). Triphenylphosphonium (TPP), a highly positive lipophilic cation, was found to preferentially accumulate in the mitochondria of cells, and used as mitochondrial targeting ligand 32,33. TPP-DOX conjugate or DOX-loaded TPP-functionalized nanomedicines selectively localized in the mitochondria of cancer cells after cell culture, and showed unique ability to overcome DOX resistance 34-37. Many evidences demonstrated that TPP is powerful for *in vitro* delivery of DOX or other anticancer drugs, however, the *in vivo* applications of highly positive charged TPP-containing nanocarriers were often limited due to rapid clearance from the blood after *i.v.* injection. Our previous work also demonstrated that the strong protein adsorption on TPP-containing lipid-polymer hybrid nanoparticles (PLGA/CPT) was observed, which resulted in extremely low tumor accumulation and low anticancer efficacy* in vivo* 38. Although shielding of the positive charge of TPP on the surface of nanoparticle with poly(ethyleneglycol) chain could partly address this problem, it decreased the tumor penetration ability and mitochondrial targeting ability inside cancer cells. Based on the weakly acidic microenvironment in tumor tissue, constructing charge reversible nanoparticles by the formation of tumor acidity triggered cleavable amide bonds from amino groups and 2,3-dimethylmaleic anhydride (DMMA) has been developed as a general strategy for positively-charged nanocarriers to avoid protein adsorption and blood clearance, enhance tumor accumulation and penetration, and improve therapeutic effect* in vivo* 39,40. Different from amine, there is no suitable site in TPP to directly react with DMMA and form cleavable bond, implying the impossibility in the construction of charge reversible TPP-containing nanocarrier by direct chemical reaction.

**Scheme 1:**
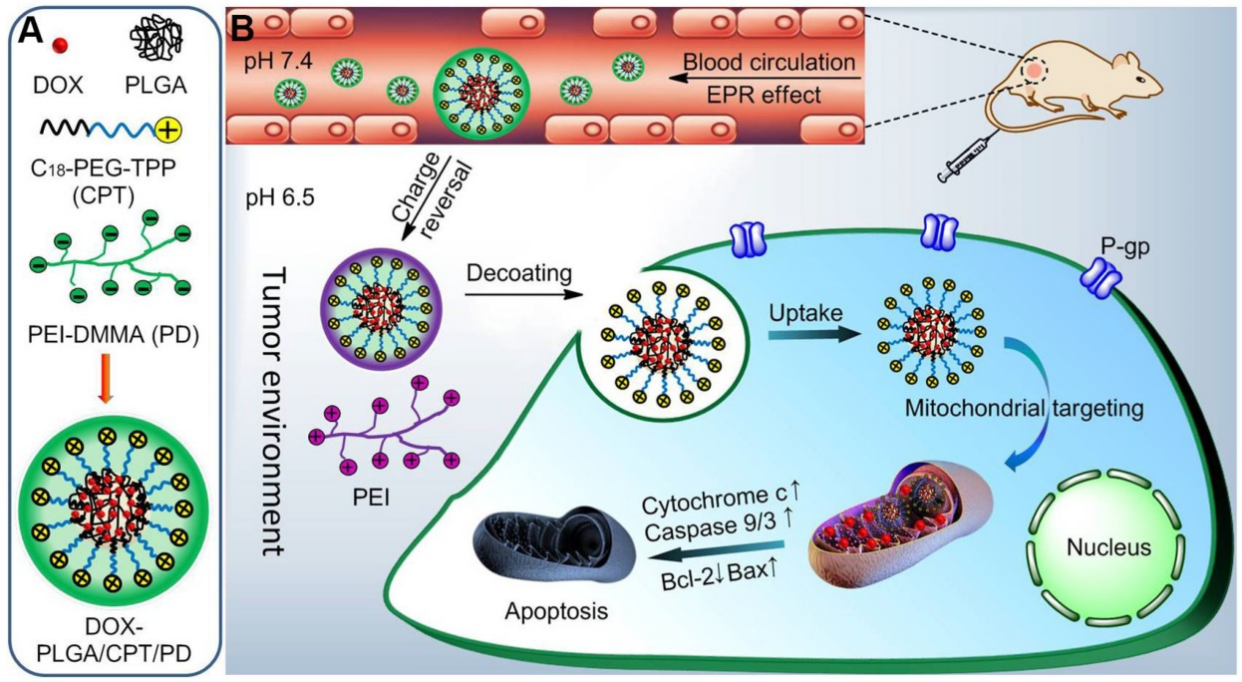
(A) Construction of DOX-loaded PLGA/CPT/PD. (B) Schematic illustration of tumor acidity triggered charge reversal and mitochondrial targeting activation of DOX-PLGA/CPT/PD to overcome drug resistance.

We here reported an indirect strategy to develop a charge reversible TPP-based nanocarrier for sequential enhancement of tumor accumulation, tumor penetration, mitochondrial targeting and overcoming DOX resistance of MCF-7/ADR breast cancer (Scheme 1). Negatively charged PEI-DMMA (PD), synthesized from branched polyethylenimine (PEI, Mw 1800 Da) and DMMA, was coated on the surface of positively charged TPP-containing lipid-polymer hybrid nanoparticles (DOX-PLGA/CPT) *via* electrostatic interaction to form DOX-PLGA/CPT/PD. The surface charge of DOX-PLGA/CPT/PD is negative and stable at neutral pH and in the blood to avoid the adsorption of serum protein and enhance tumor accumulation. Due to the tumor pH (6.5-6.8) triggered cleavage characteristic of amide bonds in PD, PD is hydrolyzed in the tumor tissue and converted to PEI and DMMA. The detachment of PEI layer, due to the loss of electrostatic force between PEI and DOX-PLGA/CPT, results in the conversion of DOX-PLGA/CPT/PD into DOX-PLGA/CPT. Thus obtained positively charged DOX-PLGA/CPT is beneficial for cancer cell uptake and localization in the mitochondria of cancer cells. This kind of weakly acidic pH activated mitochondrial targeting delivery of DOX showed excellent ability to reverse the DOX resistance of MCF-7/ADR.

## Results and discussion

### Preparation and characterization of LPNPs and DOX loaded LPNPs

The syntheses of PEI-DMMA (PD) from PEI and 2,3-dimethylmaleic anhydride (DMMA), and PEI-SA (PS) from PEI and succinic anhydride (SA) *via* one step amidation of amine with anhydride are shown in Scheme S1. The ^1^H NMR (Figure S1-S2) and FT-IR spectra (Figure S3) confirmed the chemical structures of PD and PS. PLGA/CPT, mitochondrial targeting lipid-polymer hybrid nanoparticles (LPNP), was prepared from PLGA and C_18_-PEG_2000_-TPP (CPT) using a modified nanoprecipitation method we previously described 38. PLGA/CPO as a non-mitochondrial targeting LPNP control was similarly prepared from PLGA and C_18_-PEG_2000_-OH (CPO). Afterward, the negatively charged PD and PS were separately coated on the surface of positively charged PLGA/CPT *via* electrostatic interaction to form negatively charged PLGA/CPT/PD and PLGA/CPT/PS, respectively. DOX was added to prepare DOX-PLGA/CPO, DOX-PLGA/CPT, DOX-PLGA/CPT/PD and DOX-PLGA/CPT/PS. The drug loading contents (DLC) were ranging from 5.39% to 6.10%.

**Figure 1:**
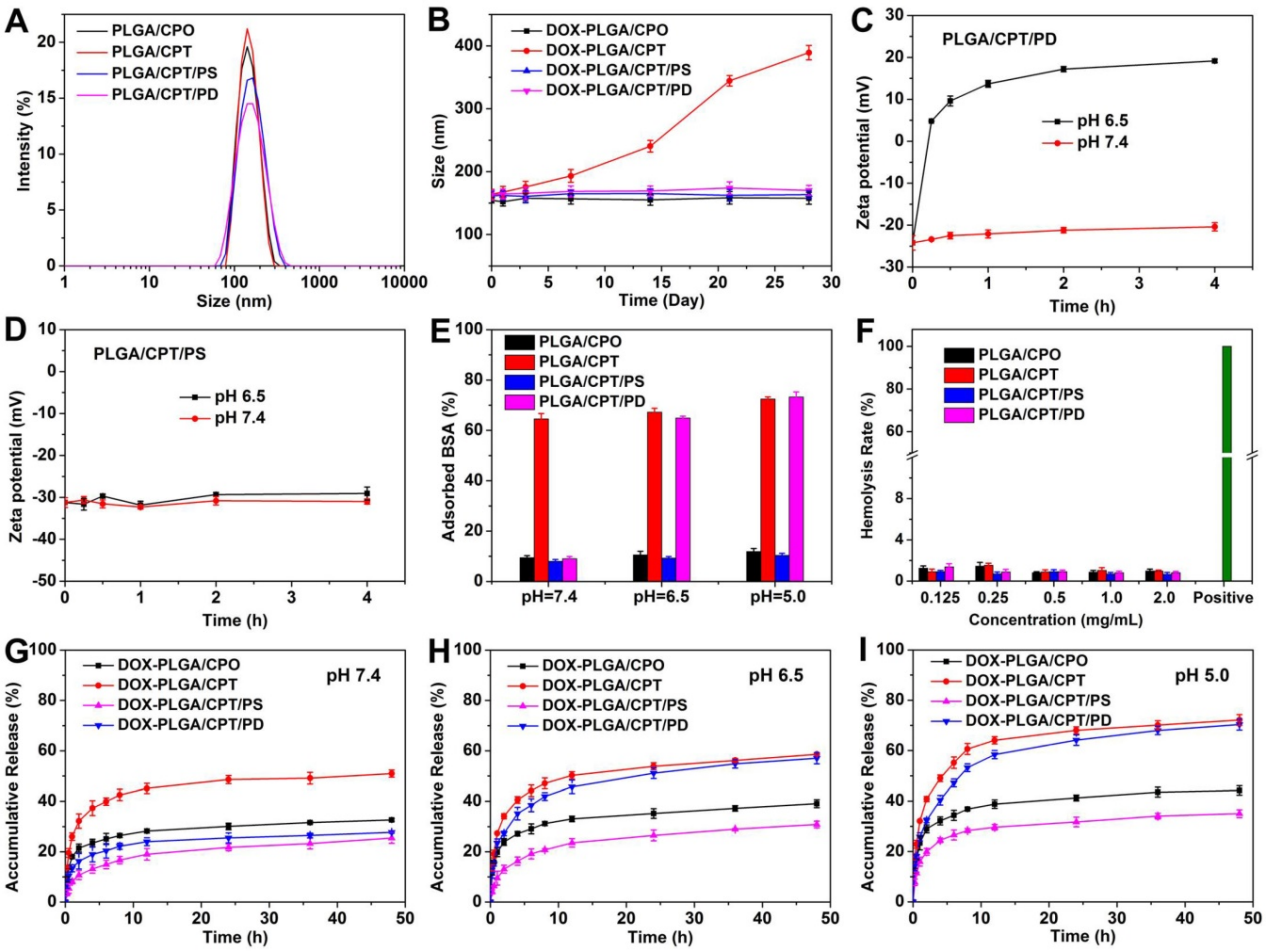
(A) Hydrodynamic sizes of LPNPs in PBS. (B) Colloidal stability of DOX-LPNPs stored at 4 °C. (C-D) Zeta potential changes of PLGA/CPT/PD and PLGA/CPT/PS incubated at pH 7.4 and pH 6. (E) BSA adsorption of LPNPs at pH 7.4, pH 6.5 and pH 5.0. (F) Hemolysis rates of LPNPs. (G-I) *In vitro* drug release of DOX-LPNPs at pH 7.4, pH 6.5 and pH 5.0.

As shown in Figure 1A and Table S1, the hydrodynamic average sizes of PLGA/CPO and PLGA/CPT in PBS determined with DLS analysis were 146 and 149 nm, respectively, which were consistent with the TEM observations (Figure S4). The coating of PLGA/CPT with PD or PS led to slight increase of average size with surface charge changing from highly positive to highly negative. Besides, when incubated in RPMI 1640 medium containing 10% fetal bovine serum (FBS) for 2 h, the hydrodynamic size of PLGA/CPT was significatly increased (319.2 nm), while negligible effects of FBS on the sizes of PLGA/CPO, PLGA/CPT/PS, and PLGA/CPT/PD were observed (Figure S5). Only slight effects of DOX-loading on average sizes and zeta potentials of LPNPs were observed (Figure S6).

All DOX-free and DOX-loaded LPNPs were well dispersed with low polydispersity indexes (PDIs). In addition, as shown in Figure 1B and Figure S7, DOX-PLGA/CPO, DOX-PLGA/CPT/PS and DOX-PLGA/CPT/PD were colloidally stable with no significant changes of average sizes and PDIs after storage at 4 °C even for four weeks. However, DOX-PLGA/CPT can only be stable for 3-day storage at 4 °C. Both the average size and PDI of DOX-PLGA/CPT gradually increased after further storage. The results demonstrated that coating DOX-PLGA/CPT with negatively charged PD or PS can greatly improve the colloidal stability.

### Acidity-triggered charge reversal

It is known that surface charge of nanomedicine plays an important role in drug delivery. Negatively charged nanomedicine is favorable to prolong the circulation lifetime in the blood and enhance tumor accumulation, but disadvantageous to cancer cell uptake. It is ideal for a nanomedicine to be negatively charged in the process of blood circulation, and converted to be positively charged in the tumor tissue. Tumor-acidity-cleavable maleic acid amide (TACMAA) provides the possibility of charge reversal of a nanomedicine from negative to positive under the weakly acidic environment of tumor tissue. The design of traditional charge reversible nanomedicine is based on tumor acidity triggered hydrolysis of amide and conversion into amine. The protonation of amine provides the positive charge after the hydrolysis of amide. In the case of PLGA/CPT/PD, the amide in PD is stable at pH 7.4 and cleavable at weakly acidic pH. We predicted that the hydrolysis of amide in PD occurred when treated at pH 6.5. The loss of electrostatic force between thus formed PEI and PLGA/CPT resulted in the deshielding of coating layer to form coating-free PLGA/CPT and expose the TPP positive charges on the surface of nanoparticles. The results verified the above hypothesis. As shown in Figure 1C, no significant changes in the zeta potentials of PLGA/CPT/PD were observed when incubated in PBS buffer 7.4 for 4 h. However, the zeta potentials of nanoparticles increased rapidly from -24.2 mV to +4.8 mV within the first 15 min, and increased to +19.2 mV after incubation for 4 h in PBS 6.5. In addition, the acidic treatment of PLGA/CPT/PD did not lead to size increase or nanoparticles aggregation (Figure S8), which is important for efficient uptake by cancer cells in tumor tissue. In contrast, unlike acid-hydrolysable PD, the amide bonds in PS are stable enough under mild acid environments. PLGA/CPT/PS exhibited a negative charge at about -30 mV with almost no variation after 4 h incubation in both PBS 7.4 and 6.5 (Figure 1D), indicating a charge irreversible characteristic of PLGA/CPT/PS. Superior to amine-based charge reversible nanomedicine, TPP-based charge reversible nanomedicine is expected to be advantageous to both cellular uptake and intracellular mitochondrial targeting.

### Protein adsorption

The electrostatic adsorption of anionic proteins by cationic nanoparticles during blood circulation always leads to rapid clearance by reticuloendothelial system (RES). It is necessary for cationic nanoparticles to avoid protein adsorption in the blood. We used bovine serum albumin (BSA) as a model protein to evaluate the interaction between LPNPs (0.5 mg/mL) and protein (0.25 mg/mL). As shown in Figure 1E, after co-incubation for 2 h at pH 7.4, all of the negatively charged nanoparticles PLGA/CPO, PLGA/CPT/PS and PLGA/CPT/PD, showed weak BSA adsorption ability. Less than 10% of BSA was separately adsorbed by PLGA/CPO, PLGA/CPT/PS and PLGA/CPT/PD. For PLGA/CPT with highly positive charges, 65% of BSA was adsorbed. The results indicated that coating of PLGA/CPT with anionic PD or PS efficiently inhibited the interaction with BSA and improved the protein-resistance. It was further found that the ability of PLGA/CPT/PD to adsorb protein was recovered when mixed together with BSA at pH 6.5 or pH 5.0. Differently, no significant differences in the adsorption of BSA by PLGA/CPT/PS were observed when the solution pH changed from 7.4 to 6.5 or 5.0. The protein adsorption results were consistent with the different hydrolysis characteristics of polyanions in PLGA/CPT/PD and PLGA/CPT/PS at different pH.

### Hemolysis assay

To evaluate the blood compatibility of LPNPs, hemolysis rates were measured using the red blood cells (RBC) of SD mice. As shown in Figure 1F, all of the LPNPs at tested concentrations exhibited relatively low hemolysis rates (< 2%). The optical photographs of the treated samples after centrifugation (Figure S9) further intuitively confirmed the excellent blood compatibility of LPNPs. It could be observed that the RBC of the positive control group (ultrapure water) exhibited obvious hemolysis, while the RBC treated by LPNPs were almost as intact as the negative control (saline). Thus, these LPNPs have great potential for *in vivo* applications.

### *In vitro* drug release

The important task of a mitochondrial targeting delivery system is to carry sufficient amount of drug into the mitochondria of cells. It is essential for a mitochondrial targeting nanomedicine to avoid drug leakage or drug release before entering into the mitochondria and release the loaded drug once localize in the mitochondria. We separately measured the release of DOX from all DOX-loaded LPNPs in PBS buffer at pH 7.4, 6.5 and 5.0. As shown in Figure 1G-I, at pH 7.4, the release of DOX from DOX-PLGA/CPO, DOX-PLGA/CPT/PS and DOX-PLGA/CPT/PD were slower than that of DOX-PLGA/CPT. Due the protonation of amino group in DOX and increase of hydrophilicity at pH 6.5 or 5.0, drug release from all DOX-loaded LPNPs was accelerated by decreasing pH value of the external buffer. Due to the hydrolysis of PD and detachment from the nanoparticles, the effect of pH on the release of drug from DOX-PLGA/CPT/PD was more notable than other LPNPs. At lower pH, the release of DOX from DOX-PLGA/CPT/PD was almost same as that of DOX-PLGA/CPT. In spite of the relatively fast release of DOX from DOX-PLGA/CPT/PD at pH 6.5 and 5.0, the total release of DOX from DOX-PLGA/CPT/PD was low within the early several hours, which ensured carrying sufficient amount of DOX into the mitochondria of cancer cells.

### Cellular uptake and mechanism analysis

**Figure 2:**
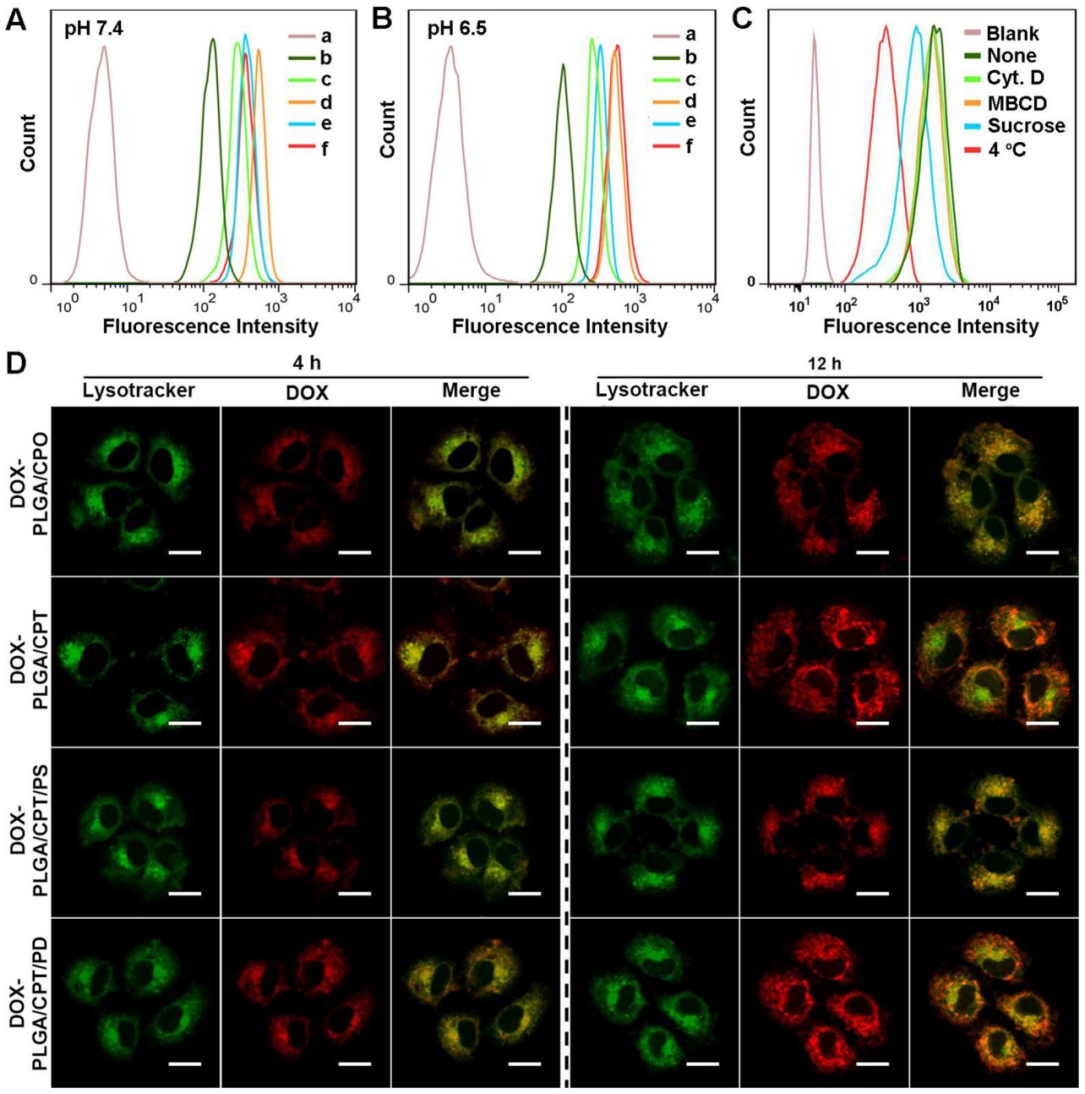
(A-B) Flow cytometry analysis of DOX fluorescence in MCF-7/ADR cells after treatment with DOX formulations for 24 h at pH 7.4 or pH 6.5 (a, PBS; b, free DOX; c, DOX-PLGA/CPO; d, DOX-PLGA/CPT; e, DOX-PLGA/CPT/PS; f, DOX-PLGA/CPT/PD). (C) Cellular uptake mechanism analysis of DOX-PLGA/CPT/PD in MCF-7/ADR cells at pH 6.5. (D) CLSM images of MCF-7/ADR cells after incubation with DOX-LPNPs for 4 h or 12 h at pH 6.5, scale bar = 20 μm.

The cellular uptake of free DOX and DOX-LPNPs at pH 7.4 or pH 6.5 was quantitatively analyzed with flow cytometry (FCM). As shown in Figure 2A and Figure 2B, due to drug efflux effect, the fluorescence intensity in free DOX-treated MCF-7/ADR cells was far lower than that of DOX-LPNPs groups. When DOX was encapsulated into the nanocarriers, the efflux effect was hindered to some extent in DOX-resistance cells, enhancing DOX accumulation in cells. Moreover, positively charged nanoparticles showed high ability to enter into cells. High fluorescence intensity of DOX was detected in MCF-7/ADR cells treated with DOX-PLGA/CPT at both pH 7.4 and 6.5. The negative charges of DOX-PLGA/CPT/PD at pH 7.4 and DOX-PLGA/CPT/PS at pH 7.4/6.5 were disadvantageous to cellular uptake. The reversal of charges from negative to positive on the surface of DOX-PLGA/CPT/PD at pH 6.5 facilitated the uptake by cells.

Next, the cellular uptake mechanism of DOX-PLGA/CPT/PD in MCF-7/ADR cells at pH 7.4 or pH 6.5 was investigated via pretreatment with endocytosis inhibitors or incubation at 4 °C, which could disturb the production of ATP in cells. Cytochalasin D (Cyt. D), methyl-β-cyclodextrin (MBCD), and sucrose were typically applied to inhibit macropinocytosis, caveolae-mediated endocytosis, and clathrin-mediated endocytosis, respectively. As shown in Figure S10 and Figure 2C, pretreatment with Cyt. D or MBCD had almost no effect on the uptake of DOX-PLGA/CPT/PD. In contrast, after pretreatment with sucrose, the uptake of DOX-PLGA/CPT/PD was obviously decreased at both pH 7.4 and pH 6.5. Moreover, incubation at a lower temperature (4 °C) gave rise to significantly reduction of the cellular uptake. Therefore, it could be concluded that the uptake pathway of DOX-PLGA/CPT/PD in MCF-7/ADR cells was mainly energy-dependent and clathrin-mediated endocytosis.

### Intracellular distribution study

After uptake by the cells, nanoparticles will be trapped by lysosomes. Whether could they escape from lysosomes greatly influence their delivery efficiency. It has been reported that the high positive charges of nanoparticles could facilitate their lysosome escape 24. To evaluated the ability of DOX-LPNPs to escape from lysosomes, MCF-7/ADR cells were stained with Lysotracker Green after 4 h or 12 h treatment at pH 6.5, and observed with confocal laser scanning microscopy (CLSM). As shown in Figure 2D, after treatment for 4 h, obvious yellow color in the merged images of DOX and Lysotracker Green could be observed in all of the DOX-LPNPs treated groups with Pearson's correlation coefficients higher than 0.8 (Figure S12), implying that most of the DOX-LPNPs were trapped by lysosomes after 4 h incubation.

When further incubated for 12 h, the red fluorescence of DOX in DOX-PLGA/CPO and DOX-PLGA/CPT/PS groups remained well overlapped with Lysotracker Green. However, the overlap ratio of these two fluorescences in DOX-PLGA/CPT and DOX-PLGA/CPT/PD groups decreased significantly with Pearson's correlation coefficients less than 0.5, demonstrating that the positively charged DOX-PLGA/CPT and charge-reversed DOX-PLGA/CPT/PD could well escape from lysosomes after 12 h incubation at pH 6.5. Due to the acidity of endosomes and lysosomes in the cells (pH 5.0-6.0), which could reverse the charges of DOX-PLGA/CPT/PD from negative to positive, similar results were observed when incubated at pH 7.4 (Figure S11).

**Figure 3:**
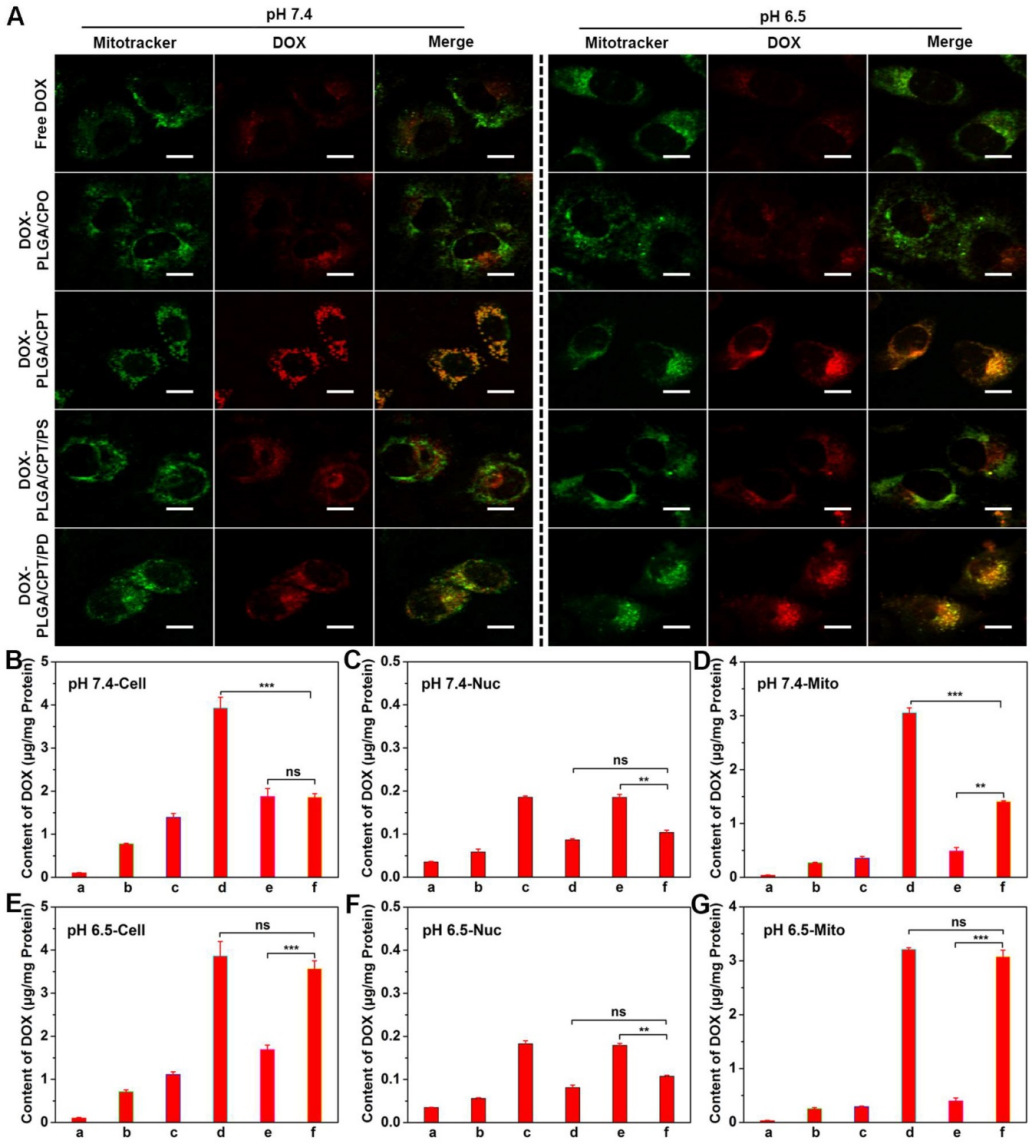
(A) CLSM images of MCF-7/ADR cells for co-localization of DOX and mitochondria, scale bar = 20 μm. (B-G) DOX contents in the whole cells, nuclei (Nuc), and mitochondria (Mito) at pH 7.4 or pH 6.5 after various treatments. a, PBS; b, free DOX; c, DOX-PLGA/CPO; d, DOX-PLGA/CPT; e, DOX-PLGA/CPT/PS; f, DOX-PLGA/CPT/PD. Data are presented as mean ± S.D (n=3). Non-significant (ns), P > 0.05, **P < 0.01, ***P < 0.001.

To further investigate the mitochondrial targeting ability of DOX-LPNPs in DOX-resistant cells, MCF-7/ADR cells were separately treated with free DOX and DOX-LPNPs at pH 7.4 or pH 6.5 for 24 h, stained with Mitotracker Green and observed with CLSM (Figure 3A). In the cells separately treated with free DOX and DOX-PLGA/CPO at either pH 7.4 or 6.5, very low overlap between green fluorescence and red fluorescence with Pearson's correlation coefficient less than 0.5 was observed, which implied low ability of free DOX and DOX-LPNPs without TPP to localize in the mitochondria. At either pH 7.4 or 6.5, DOX-PLGA/CPT treated group displayed excellent mitochondrial targeting ability with Pearson's correlation coefficient of 0.926 (Figure S13). The pH of cell culture medium showed no effect on the mitochondrial targeting ability of DOX-PLGA/CPT. In the cases of both DOX-PLGA/CPT/PS and DOX-PLGA/CPT/PD, the positive charge of TPP was shielded by negative charge of PS or PD. The hydrolysis ability of amide bonds in PS and PD determined the mitochondrial targeting ability of DOX-PLGA/CPT/PS and DOX-PLGA/CPT/PD. The amide bonds in PS are not cleavable at both pH 7.4 and 6.5. The positive charges and mitochondrial targeting ability of TPP in DOX-PLGA/CPT/PS are fully hided and cannot be recovered due to the strong binding of PS to TPP even after uptake by MCF-7/ADR cells. At both pH 7.4 and 6.5, DOX-PLGA/CPT/PS showed very low fluorescence overlap with Pearson's correlation coefficient less than 0.5. The mitochondrial targeting characteristic of DOX-PLGA/CPT/PD is different from that of DOX-PLGA/CPT/PS. DOX-PLGA/CPT/PD showed relatively high mitochondrial targeting with Pearson's correlation coefficient of 0.815 in MCF-7/ADR cells co-cultured in pH 7.4 medium. After entry into the cells, the acidity of endosomes and lysosomes in the cells (pH 5.0-6.0) triggered the hydrolysis of PD in DOX-PLGA/CPT/PD led to formation of DOX-PLGA/CPT and recovery of mitochondrial targeting ability. Higher mitochondrial targeting ability of DOX-PLGA/CPT/PD with Pearson's correlation coefficient of 0.911 was observed when co-cultured with MCF-7/ADR cells at pH 6.5, which was comparable to DOX-PLGA/CPT (0.926), demonstrating full activation of mitochondrial targeting ability of DOX-PLGA/CPT/PD. Part or full hydrolysis of amide in PD in pH 6.5 cell culture medium is advantageous to recover the mitochondrial targeting of DOX-PLGA/CPT/PD.

The DOX contents in the whole cells, nuclei, and mitochondria of MCF-7/ADR cells were further quantitatively measured after separate treatment for 24 h with free DOX and DOX-LPNPs at a DOX concentration of 10 μg/mL. As shown in Figure 3B and Figure 3E, the results of DOX contents measurement in the whole cells treated with various DOX formulations were consistent with that of FCM analysis and CLSM observation. Only a small amount of DOX could be detected in the nuclei of all groups both at pH 7.4 and pH 6.5 (Figure 3C and Figure 3F), and the DOX content in the nuclei of DOX-PLGA/CPT or DOX-PLGA/CPT/PD treated cells exhibited less than other groups. In addition, DOX contents in the mitochondria of MCF-7/ADR cells separately treated with free DOX, DOX-PLGA/CPO and DOX-PLGA/CPT/PS at both pH 7.4 and 6.5 were negligible. High contents of DOX were detected in MCF-7/ADR cells treated with DOX-PLGA/CPT at both pH 7.4 and pH 6.5 (Figure 3D and Figure 3G). At pH 7.4, DOX contents in whole cells and mitochondria of MCF-7/ADR cells treated with DOX-PLGA/CPT/PD were significantly lower than that of DOX-PLGA/CPT treated cells, however, there were almost no differences between cells treated with DOX-PLGA/CPT/PD and DOX-PLGA/CPT at pH 6.5. The measurement of DOX contents in whole cells and mitochondria provided more solid evidences to confirm that the weak acidity triggered charge reversal of DOX-PLGA/CPT/PD improved cellular uptake and realized mitochondrial targeting.

### *In vitro* cytotoxicity

**Figure 4:**
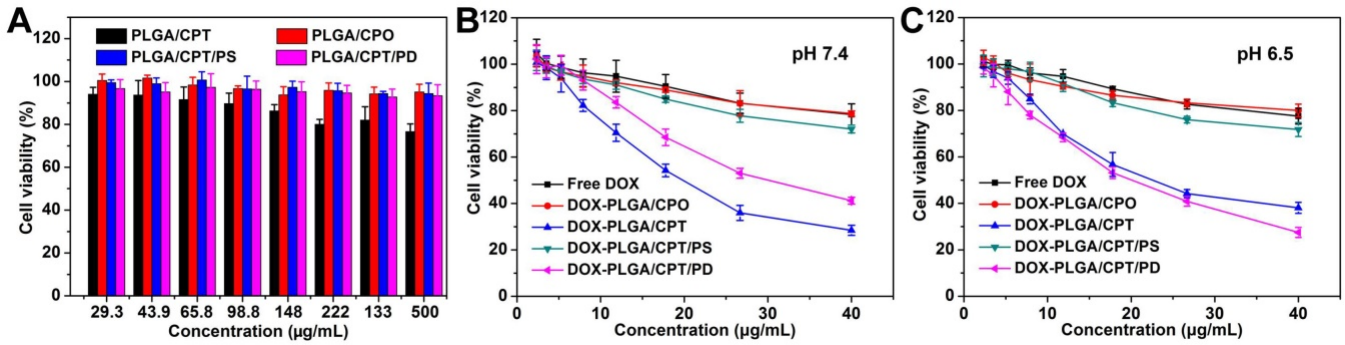
(A) Cytotoxicity of LPNPs against MCF-7/ADR cells. (B-C) Cytotoxicity of DOX formulations against MCF-7/ADR cells at pH 7.4 and pH 6.5.

MTT assay was used to evaluate the cytotoxicity of LPNPs and DOX-loaded LPNPs against MCF-7/ADR cells or COS 7 cells. As shown in Figure 4A and Figure S14, for all of the LPNPs without loading DOX, only PLGA/CPT exhibited slight toxicity to MCF-7/ADR cells and COS 7 cells. All other LPNPs did not show notable toxicity even at a high concentration of 500 μg/mL. The cytotoxicity results of DOX-containing formulations were shown in Figure 4B and Figure 4C. Either at pH 7.4 or pH 6.5, free DOX, DOX-PLGA/CPO and DOX-PLGA/CPT/PS only showed low toxicity against MCF-7/ADR cells at the tested concentration (40 μg/mL) and were incapable to overcome DOX resistance of MCF-7/ADR cells. At both pH 7.4 and pH 6.5, DOX-PLGA/CPT efficiently reversed DOX resistance of MCF-7/ADR cells with IC_50_ of 19.82 μg/mL and 22.60 μg/mL, respectively. At pH 7.4, although the cellular uptake of DOX-PLGA/CPT/PD was inhibited by the negative charges, it still showed moderate toxicity to MCF-7/ADR cells with IC_50_ of 30.13 μg/mL due to the charge-reversal and recovery of mitochondrial targeting ability after escape from acid endosomes and lysosomes. The ability of DOX-PLGA/CPT/PD to overcome DOX resistance of MCF-7/ADR cells (IC_50_ 19.82 μg/mL) was greatly improved at pH 6.5, which was ascribed to the charge reversal of DOX-PLGA/CPT/PD under weakly acidic conditions and thus promoted its cellular uptake. Although both DOX-PLGA/CPT and DOX-PLGA/CPT/PD can overcome the drug resistance of MCF-7/ADR cells *in vitro*, due to the highly positive charge nature of DOX-PLGA/CPT, it is expected that DOX-PLGA/CPT/PD is superior for *in vivo* application.

### Mitochondrial damage and apoptosis mechanism analysis

**Figure 5:**
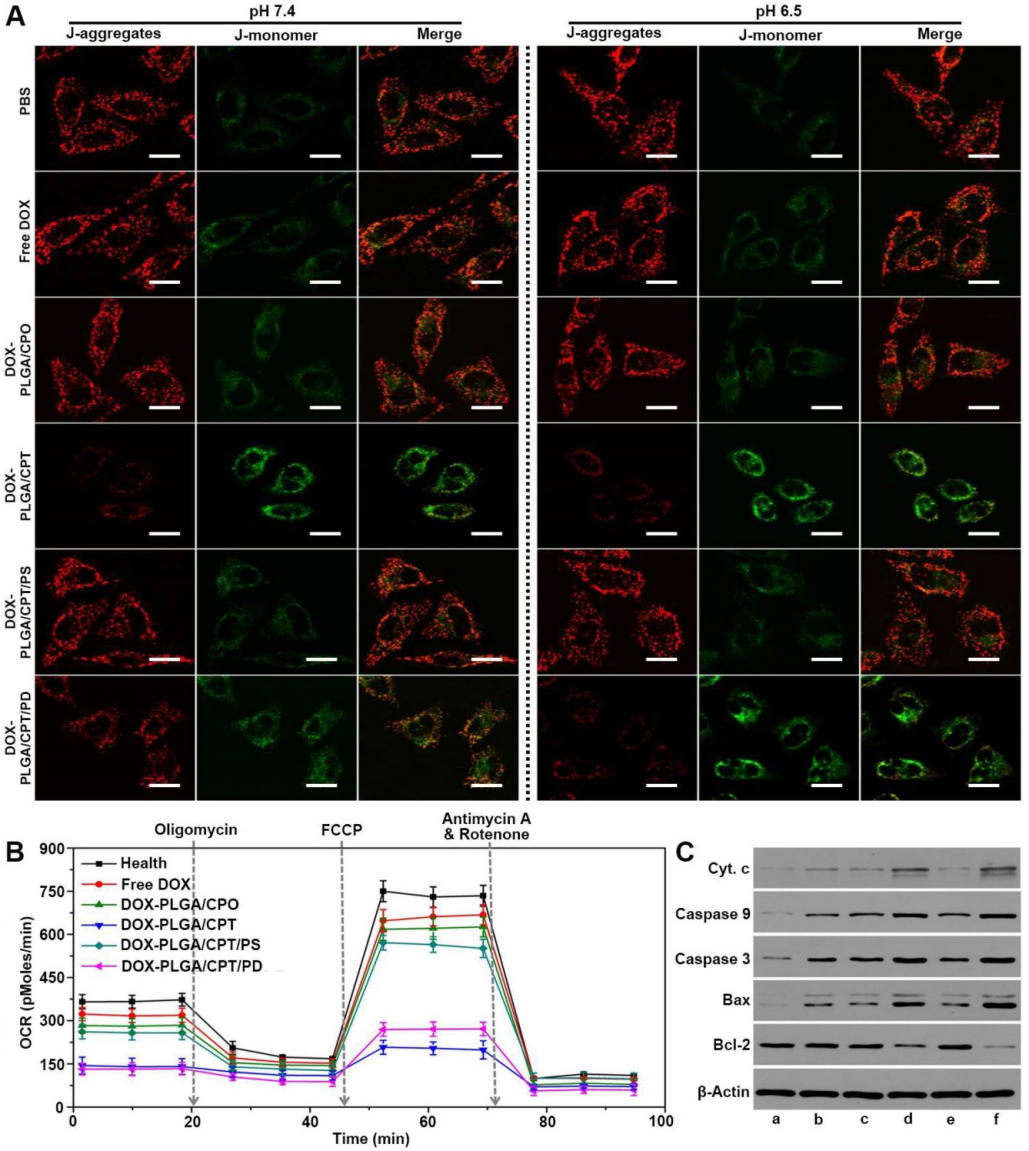
(A) CLSM images of MCF-7/ADR cells stained with JC-1 after treatment with DOX formulations for 24 h, scale bar = 20 μm. (B) Oxygen consumption rate (OCR) of MCF-7/ADR cells after treatment with or without DOX formulations at pH 6.5 for 24 h. (C) Western blot analysis of apoptosis-related proteins after various treatments at pH 6.5 for 24 h, a, PBS; b, free DOX; c, DOX-PLGA/CPO; d, DOX-PLGA/CPT; e, DOX-PLGA/CPT/PS; f, DOX-PLGA/CPT/PD. The concentration of DOX was 20 μg/mL.

JC-1, a dye aggregating in the normal mitochondria to emit red fluorescence and inversely dispersing into a monomeric form to emit green fluorescence when the mitochondrial membrane potential (∆Ψ_m_) decreased, was used to evaluate mitochondria damage of cancer cells. As shown in Figure 5A, strong red fluorescence and weak green fluorescence were observed after separate treatment with PBS, free DOX, DOX-PLGA/CPO and DOX-PLGA/CPT/PS for 24 h either at pH 7.4 or at pH 6.5, demonstrating relatively healthy mitochondria of MCF-7/ADR cells in these groups. In contrast, when the cells were treated with mitochondria-targeting DOX-PLGA/CPT, there was weak red fluorescence and strong green fluorescence, suggesting severe damage of mitochondria. The treatment of cells with DOX-PLGA/CPT/PD at pH 7.4 resulted in moderate mitochondrial damage with relatively weak red fluorescence and green fluorescence of JC-1. At pH 6.5, the observation results of red fluorescence and green fluorescence in DOX-PLGA/CPT/PD treated cells were almost same as that in DOX-PLGA/CPT treated cells, demonstrating the same ability of DOX-PLGA/CPT and DOX-PLGA/CPT/PD to disrupt the mitochondria.

Afterward, Seahorse XF24 extracellular flux analyzer was used to analyze the mitochondrial bioenergetics and evaluate the mitochondrial functions of MCF-7/ADR cells treated with DOX formulations at pH 6.5. Oxygen consumption rate (OCR) is an important marker of OXPHOS. As shown in Figure 5B, consistent with the results in the cytotoxicity assay at pH 6.5, in comparison to healthy cells without any treatment, the treatment of MCF-7/ADR cells with free DOX, DOX-PLGA/CPO, or DOX-PLGA/CPT/PS resulted in only slight change in the basal oxygen consumption rate (OCR). The basal OCR of MCF-7/ADR cells separately treated with DOX-PLGA/CPT and DOX-PLGA/CPT/PD were significantly lower than that of healthy cells. Subsequently, oligomycin, FCCP, and rotenone/antimycin-A were successively added to modulate mitochondrial respiration and measure maximal glycolytic capacity, maximum OXPHOS respiration capacity and non-mitochondrial respiration of the cells, respectively. The basal respiration, ATP turnover, maximum respiration rate, spare respiratory capacity, coupling efficiency, and electron transport chain (ETC) accelerator response of healthy and DOX-formulations treated MCF-7/ADR cells were calculated from the OCR analysis. It was found that both DOX-PLGA/CPT and DOX-PLGA/CPT/PD treatment led to more significant decrease in ATP turnover, maximum respiration rate, spare respiratory capacity, coupling efficiency and electron transport chain (ETC) accelerator response and more grievous injury to the mitochondria than that of free DOX, DOX-PLGA/CPO and DOX-PLGA/CPT/PS treatment groups (Figure S15).

To further characterize the death pathway of MCF-7/ADR cells treated with DOX formulations, the expressions of apoptosis-related proteins were detected using western blot analysis. As shown in Figure 5C, compared with the control group of PBS treatment, the expressions of cytochrome c, caspase-9, caspase-3 and Bax were upregulated in all of MCF-7/ADR cells incubated with various DOX-containing formulations at pH 6.5, however, both of DOX-PLGA/CPT and DOX-PLGA/CPT/PD treatments resulted in higher expressions of these pro-apoptotic proteins than that of free DOX, DOX-PLGA/CPO and DOX-PLGA/CPT/PS groups. On the other hand, the expressions of antiapoptotic protein Bcl-2 in MCF-7/ADR cells were significantly inhibited by DOX-PLGA/CPT and DOX-PLGA/CPT/PD. Free DOX, DOX-PLGA/CPO and DOX-PLGA/CPT/PS did not significantly affect Bcl-2 protein expression in MCF-7/ADR cells. Taking together, it can be concluded that the mitochondria-dependent apoptosis is at least an important pathway for death of MCF-7/ADR cells treated with mitochondrial targeting delivery of DOX-PLGA/CPT and DOX-PLGA/CPT/PD.

### Pharmacokinetics and biodistribution of DOX *in vivo*

**Figure 6:**
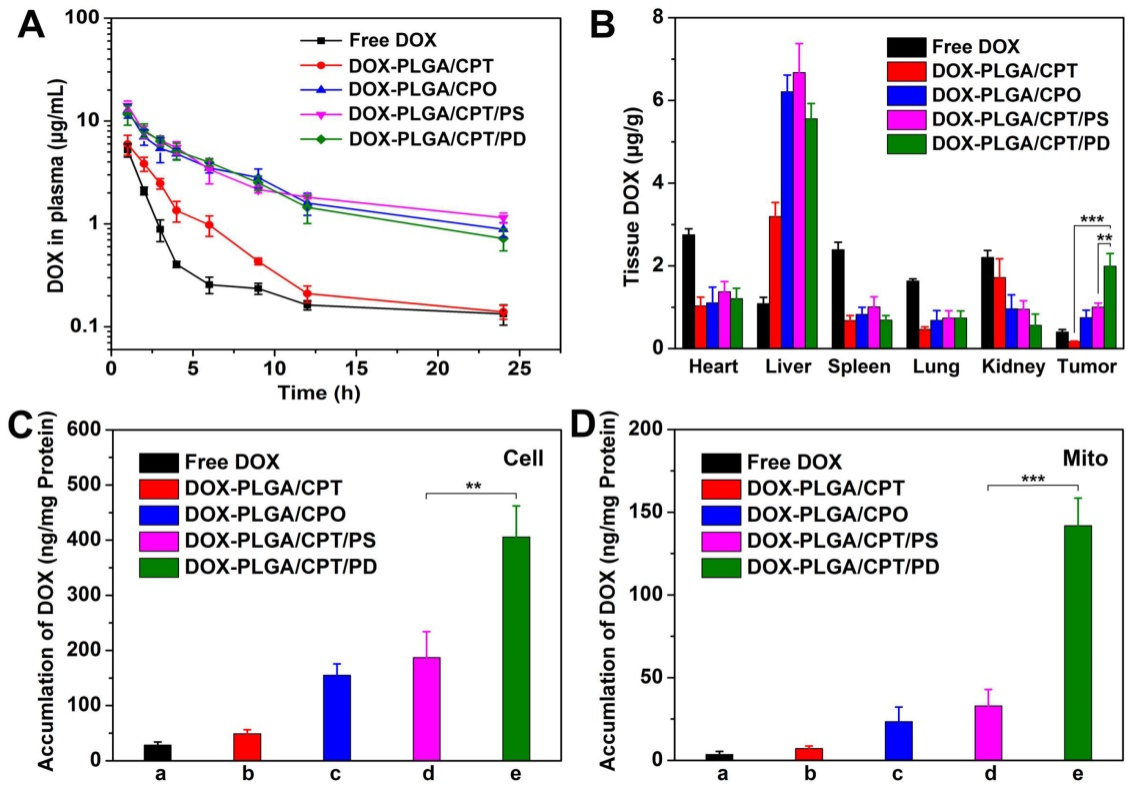
(A) Blood circulation of free DOX and DOX-LPNPs after intravenous injection. (B) Biodistribution of DOX in MCF-7/ADR tumor-bearing mice at 24 h after intravenous injection. (C-D) Quantitative analysis of DOX contents in the whole tumor cells and the mitochondria of tumor cells at 24 h after intravenous injection.

Prior to evaluate the *in vivo* therapeutic effects of DOX-containing formulations, we first investigated the pharmacokinetics of free DOX and DOX-LPNPs. We assumed that DOX-PLGA/CPT with highly positive charges will adsorb a lot of serum proteins and be cleared rapidly. The results shown in Figure 6A and Table S2 confirmed this hypothesis. After intravenous injection, free DOX and DOX-PLGA/CPT were rapidly cleared in the blood circulation with half-life time (t_1/2Z_) 2.71 h and 4.13 h, respectively. As a contrast, DOX-PLGA/CPO, DOX-PLGA/CPT/PS, and DOX-PLGA/CPT/PD showed significantly slower clearance with t_1/2Z_ 16.92 h, 18.82 h, and 15.84 h, respectively. Therefore, pegylated or polyanion coated nanoparticles with negative charges could avoid the rapid clearance in blood circulation.

The prolongation of blood circulation time was expected to facilitate the accumulation of nanoparticles in tumor site *via* EPR effect. Next, we analyzed the biodistribution of DOX in the main tissues (heart, liver, spleen, lung, kidney, and tumor) of MCF-7/ADR tumor-bearing nude mice at 24 h after intravenous injection. The collected tissues were fluorescently imaged using a living image IVIS^®^ spectrum (Figure S16). Very weak fluorescence was observed in the tumor tissue of nude mice treated with DOX-PLGA/CPT. Moderate strength fluorescence in tumor tissue were detected after separate injection of DOX-PLGA/CPO and DOX-PLGA/CPT/PS. The injection of DOX-PLGA/CPT/PD resulted in the strongest fluorescence in tumor tissue among the all groups. The emission ability of DOX is strongly related to its aggregation state. It is reasonable to compare the fluorescence strength in tumor tissue after injection of DOX-PLGA/CPT, DOX-PLGA/CPO, DOX-PLGA/CPT/PS, and DOX-PLGA/CPT/PD due to the similar encapsulation in nanocarriers and aggregation state of DOX in tumor tissue. Although moderate fluorescence was observed in tumor tissue after injection of free DOX, it did not imply moderate concentration of DOX in the tumor tissue due to the strong emission ability of free DOX in tumor tissue. To draw a correct and reliable conclusion, the tissues were grinded to extract DOX for quantitative determination with HPLC analysis (Figure 6B). DOX concentration in the heart after injection of free DOX is the highest among all groups. Cardiotoxicity is the most important side effect of DOX used in the clinic. The encapsulation of DOX in all nanocarriers tested in this work could significantly decrease DOX concentration in the heart and possibly decrease cardiotoxicity. In the case of free DOX injection, very low concentration of DOX in the tumor tissue was detected due to the absence of EPR effect of small molecular anticancer drug. There was negligible DOX distributed in the tumor tissue after injection of cationic DOX-PLGA/CPT, which was ascribed to the adsorption of proteins in the blood and rapid clearance of cationic nanocarrier in the blood circulation. Injection of DOX-PLGA/CPO and DOX-PLGA/CPT/PS, negatively charged and charge irreversible nanomedicine, led to moderate accumulation of DOX in the tumor tissue due to the EPR effect. DOX concentration in the tumor tissue after injection of DOX-PLGA/CPT/PD with charge reversal characteristic was almost 2 folds higher than that of DOX-PLGA/CPO and DOX-PLGA/CPT/PS. DOX-PLGA/CPO, DOX-PLGA/CPT/PS and DOX-PLGA/CPT/PD exhibited EPR effect *in vivo*. Once they reached tumor tissues, the conversion of negatively charged DOX-PLGA/CPT/PD to positively charged DOX-PLGA/CPT enhanced the interaction of nanocarriers and tumor cells, which facilitated the cellular uptake and avoided being excreted from the tumor tissue.

We further separated the tumor cells from collected tumor tissues and determined DOX contents in the tumor cells. As shown in Figure 6C, it was not surprised that extremely low concentration of DOX was detected in the tumor cells of free DOX injection group due to DOX-resistance and drug efflux effect of MCF-7/ADR. Although a part of small molecular DOX accumulated in tumor tissue, it was difficult to be internalized into tumor cells.

Different from free DOX injection group, relatively high concentration of DOX was detected in tumor cells of DOX-PLGA/CPT injection group, which indicated that cationic nanocarrier was disadvantageous to accumulation in tumor tissue but advantageous to cellular uptake. In the cases of DOX-PLGA/CPO and DOX-PLGA/CPT/PS injection groups, DOX contents in tumor cells were far higher than that of free DOX and DOX-PLGA/CPT injection groups. Similar to that in tumor tissue, the content of DOX in DOX-PLGA/CPT/PD injection group was the highest, which was 2 folds higher than that of DOX-PLGA/CPO and DOX-PLGA/CPT/PS injection groups, demonstrating that the charge reversal characteristic of DOX-PLGA/CPT/PD played an important role in improving tumor cellular uptake of the nanomedicine.

We next isolated the mitochondria from the tumor cells and measured DOX contents (Figure 6D). Extremely low concentrations of DOX were detected in the groups of free DOX, DOX-PLGA/CPT, DOX-PLGA/CPO and DOX-PLGA/CPT/PS groups. As we expected, DOX content in the mitochondria of DOX-PLGA/CPT/PD groups was the highest, which was 4.4 folds and 6.2 folds higher than that of DOX-PLGA/CPT/PS and DOX-PLGA/CPO groups, respectively. Therefore, although DOX-PLGA/CPT could work well in mitochondrial targeting *in vitro*, it was powerless *in vivo*. The perfect ability of DOX-PLGA/CPT/PD in mitochondrial targeting both *in vitro* and *in vivo* predicted the possibility in overcoming the resistance of MCF-7/ADR tumor *in vivo*.

### *In vivo* therapeutic effect

**Figure 7:**
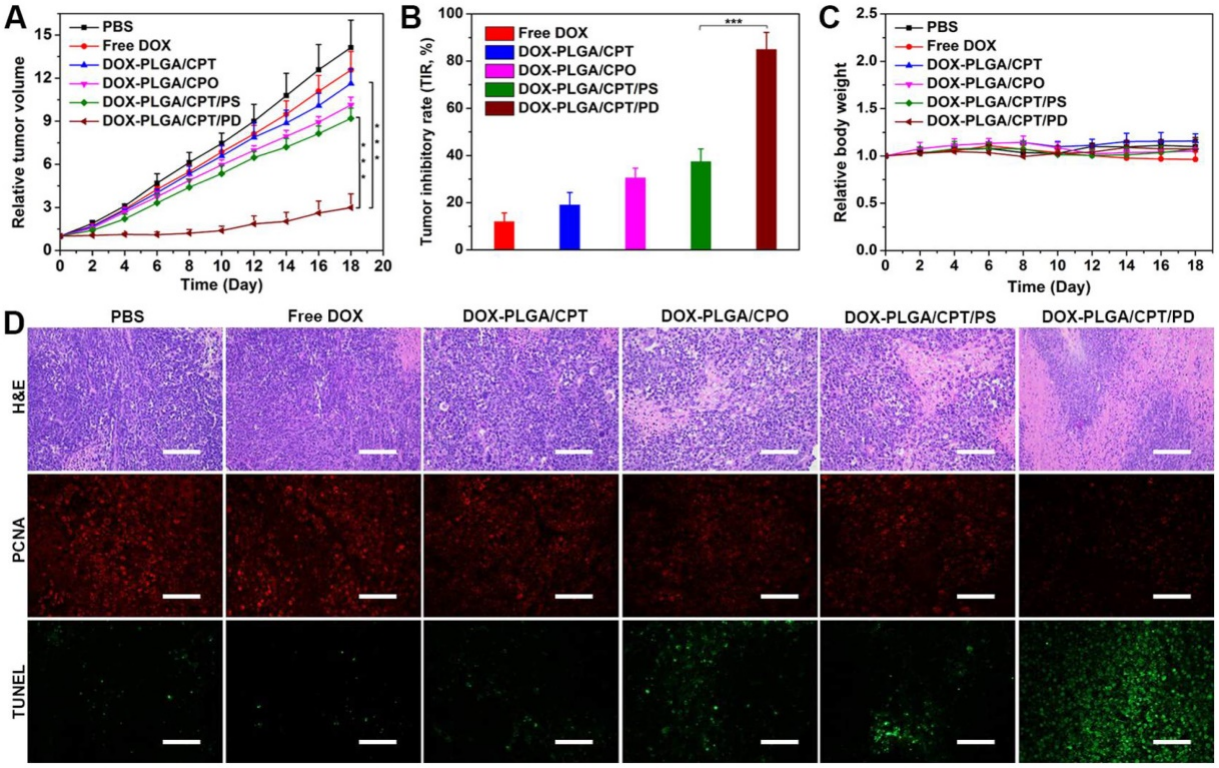
(A) The tumor growth curves of MCF-7/ADR tumor-bearing mice after various treatments. (B) Tumor inhibition rates of DOX formulations after treatment for 18 days. (C) Relative body weights of the treated mice. (D) H&E staining, PCNA and TUNEL analyses of tumor tissues after various treatments for 18 days, scale bar = 100 μm. Data are presented as mean ± S.D (n=3). **P < 0.01, ***P < 0.001.

The *in vivo* antitumor activities of free DOX and DOX-LPNPs were evaluated with MCF-7/ADR xenograft tumor-bearing female nude mice by intravenous injection each 3 days at a dose of 5 mg DOX per kg body weight. The original tumor volumes before treatments were approximately 100 mm^3^. The tumor volumes (Figure 7A) and body weights of nude mice were monitored each 2 days. At 18^th^ day after the first injection, in comparison to the control (PBS treatment), free DOX treatment showed negligible effect on inhibiting MCF-7/ADR growth with a tumor inhibition rate (TIR) of 11.9% (Figure 7B). DOX-PLGA/CPT, highly effective to inhibit MCF-7/ADR cells growth *in vitro*, showed extremely low antitumor ability *in vivo* with a TIR of 19.0%. The low antitumor activities of DOX-PLGA/CPO and DOX-PLGA/CPT/PS with TIRs of 30.5% and 37.4% were separately observed. Among all treatment groups, DOX-PLGA/CPT/PD exhibited the best antitumor ability with a TIR of 84.9%. The images of isolated tumors and the tumor weights after 18-day treatment further provided intuitive support for the antitumor activity (Figure S17-S18). The *in vivo* antitumor results were highly consistent with mitochondrial DOX content analysis results. In addition, the average body weights of mice in DOX-LPNPs treatment groups were relatively stable. Slight decrease of body weight was observed at the late stage after treatment with free DOX (Figure 7C).

Hematoxylin and eosin (H&E) staining results demonstrated the injury of the heart and cardiotoxicity of free DOX, while no significant side effects in major organs of the mice were observed after treatment with all kinds of DOX-LPNPs (Figure S19). In addition, H&E staining, PCNA and TUNEL immunofluorescence analyses of the tumors tissues were carried out to further evaluate the effect of DOX-containing formulations on the tumor tissue and tumor cells *in vivo* (Figure 7D). Consistent with the results of tumor volumes, weights and images, among all of the treatment groups, DOX-PLGA/CPT/PD exhibited the largest necrotic area (H&E), the weakest signal of proliferation (PCNA), and the strongest signal of apoptosis (TUNEL). The results strongly demonstrated that the *in vivo* mitochondrial delivery of DOX with PLGA/CPT/PD could effectively overcome DOX resistance of MCF-7/ADR, induce tumor cell apoptosis, and inhibit tumor cell proliferation and tumor growth.

## Conclusion

In summary, to address the problem of cationic TPP for the application *in vivo*, a simple strategy was developed to construct charge reversible TPP-containing nanocarriers for realizing highly efficient *in vivo* delivery of DOX into the mitochondria and overcoming DOX resistance of MCF-7/ADR tumor. The positive charge of TPP in DOX-PLGA/CPT was shielded with tumor acidity triggered cleavable polyanion (PEI-DMMA, PD) *via* electrostatic interaction to form negatively charged DOX-PLGA/CPT/PD. The negative charge nature of DOX-PLGA/CPT/PD ensured the avoidance of rapid clearance in blood circulation and efficient accumulation in tumor tissue. In weakly acidic tumor tissue, the hydrolysis of amide bonds in PEI-DMMA led to the formation of cationic PEI and loss of electrostatic force to bind TPP in the nanocarrier. The recovery of positive charge in thus formed DOX-PLGA/CPT enhanced both cellular uptake and intracellular mitochondrial localization, improved intracellular DOX concentration, especially in the mitochondria of MCF-7/ADR tumor cells. The highly efficient mitochondrial delivery of DOX *in vivo* with PLGA/CPT/PD provided a promising method for overcoming the resistance of MCF-7/ADR breast cancer. The application of this kind of charge reversible and activatable mitochondrial targeting nanocarrier will not be limited in the delivery of DOX and in the therapy of MCF-7/ADR breast cancer. We believe that it will be a general strategy for mitochondrial delivery of many hydrophobic anticancer drugs and overcoming the resistance in many kinds of cancers.

## Experimental Section

### Materials

Branched polyethylenimine (PEI, M_w_ 1800 Da) and succinic anhydride (SA) were purchased from Innochem Technology Co., Ltd. (Beijing, China). 2,3-Dimethylmaleic anhydride (DMMA), PLGA (lactide : glycolide = 65 : 35, M_w_ 40000-75000 Da), 3-(4,5-dimethylthiazol-2-yl)-2,5-diphenyltetrazolium bromide (MTT), cytochalasin D (Cyt. D), methyl-β-cyclodextrin (MBCD), sucrose, collagenase I, and collagenase IV were purchased from Sigma-Aldrich (Shanghai, Trading Co., Ltd). Polyethylene glycol monostearate (C_18_-PEG_2000_-OH, CPO) was obtained from Tokyo Chemical Industry Co., Ltd. C_18_-PEG_2000_-TPP (CPT) was synthesized as our previously reported 38. Doxorubicin hydrochloride (DOX·HCl) was provided by Aladdin Reagent (Shanghai, China). N,N-dimethyl formamide (DMF), chloroform (CHCl_3_), and isopropanol were purchased from Sinopharm Chemical Reagent Co. Ltd. (Shanghai, China). Lysotracker Green (Yeasen), Mitotracker Green (Beyotime), BCA protein assay kit (Beyotime), mitochondrial membrane potential assay kit with JC-1 (Beyotime), mitochondrial isolation kit (Beyotime), nuclei extraction kit (ExKine), XF24 extracellular flux analyzer kit (Seahorse Bioscience), and matrigel matrix (BD Biosciences) were all obtained from domestic suppliers. All other reagents and solvents were of analytical grade and used as received.

### Synthesis of PEI-DMMA (PD) and PEI-SA (PS)

2,3-Dimethylmaleic anhydride (DMMA, 2.02 g) was dissolved in DMSO (5 mL) under N_2_ atmosphere. Then branched polyethylenimine (0.90 g) in DMSO (5 mL) was added dropwise into the above solution and stirred for 24 h at room temperature. Afterward, the mixture was transferred to a dialysis tube (MWCO 1000 Da) and dialyzed against pure water for 48 h. The dialysis water was maintained at pH 7-8 adjusted with saturated NaHCO_3_ and changed every 6 h. After dialysis, the solution was filtered and freeze-dried to obtain slightly yellow solid PEI-DMMA (1.93 g, yield 74.4%). PEI-SA (PS, 1.72 g) was similarly synthesized from PEI (0.90 g) and succinic anhydride (1.60 g) with a yield of 78.6%. The ^1^H NMR and FT-IR spectra of PD and PS were separately measured with Mercury 400 MHz NMR spectrometer (Bruker, Switzerland) and FTS 6000 spectrum instrument (Bio-Rad Co. Hercules, USA).

### Preparation of lipid-polymer hybrid nanoparticles (LPNPs)

C_18_-PEG_2000_-TPP (CPT, 3mg) was dispersed in 7 mL ultrapure water and stirred at 45 °C for 30 min. Subsequently, PLGA (50 mg) dissolved in DMF (3 mL) was added dropwise into the aqueous phase above under violently stirring. After stirring for another 2 h at room temperature, the mixture was transferred into a dialysis tube (MWCO 3500 Da) to dialyze against ultrapure water for 24 h. The obtained solution of hybrid nanoparticles PLGA/CPT was filtered through a syringe filter (pore size 0.8 μm) and stored at 4 °C for usage. PLGA/CPO was similarly prepared from PLGA (50 mg) and C_18_-PEG_2000_-OH (CPO, 3mg). PLGA/CPT/PD and PLGA/CPT/PS were prepared from PLGA/CPT by separately coating with PD and PS *via* electrostatic interaction. A solution of PLGA/CPT (10 mL, 3 mg/mL) was separately added dropwise into the solution of PD or PS in ultrapure water (5 mL, 2 mg/mL) and stirred for 2 h at room temperature. The mixture was transferred into a dialysis tube (MWCO 3500 Da) to dialyze against ultrapure water for 24 h. The dialysis water was maintained at pH 7-8 adjusted with saturated NaHCO_3_ and changed every 6 h.

### Preparation of DOX Loaded LPNPs

DOX·HCl (4 mg) and triethylamine (10 μL) were added into DMF (3 mL) and stirred in the dark overnight. PLGA (50 mg) was added into the solution and stirred for another 2 h. The preparation of DOX loaded LPNPs was same as that of blank LPNPs described above except the mixed solution of DOX and PLGA was used instead of a solution of PLGA in DMF. Afterward, 1 mL of the obtained DOX-LPNPs were separately lyophilized, weighted and redissolved in DMF to detect the DOX contents using RF-5301 PC spectrofluorophotometer (SHIMADZU, Japan) (λ_ex_ = 488 nm, λ_em_ = 590 nm ) according to the corresponding calibration curve. The drug loading contents (DLCs) of DOX-LPNPs were calculated according to the following formula:

DLC (wt%) = (weight of DOX / weight of DOX-LPNPs) × 100

### Characterizations of LPNPs and DOX-LPNPs

The average sizes, polydispersity indexes (PDIs), and zeta-potentials of LPNPs and DOX-LPNPs in PBS or RPMI 1640 medium containing 10% fetal bovine serum (FBS) were measured with ZETA-SIZER Nano Series ZEN3600 (Malvern Instruments Ltd, UK). In addition, 5 μL of the diluted LPNPs were separately dropped on a copper grid with formvar film and stained with 0.2% (w/v) phosphotungstic acid for 2 min. After drying in the air, the morphologies of nanoparticles were observed using Jeol JEM-100CXII transmission electron microscopy (TEM, Tokyo, Japan) at an acceleration voltage of 100 kV. To evaluate the colloidal stability of DOX-LPNPs, the sizes and PDIs of DOX loaded nanoparticles were measured at designed time intervals (0, 1, 3, 7, 14, 21, 28 days) after storage at 4 °C.

### Effect of mildly acidic environment on sizes and zeta potentials of LPNPs

The solutions of PLGA/CPT/PD and PLGA/CPT/PS were separately diluted to 0.1 mg/mL with PBS (0.01 M, pH 7.4 or pH 6.5) and incubated at 37 °C. The zeta potential of each sample was measured at designed time intervals (0, 0.25, 0.5, 1, 2, 4 h). After incubation for 4 h, sizes of the treated nanoparticles were measured.

### Protein adsorption assay

BCA protein assay kit was used to evaluate the protein adsorption of LPNPs. Briefly, PLGA/CPO, PLGA/CPT, PLGA/CPT/PS, and PLGA/CPT/PD were diluted with PBS (0.01M, pH 7.4, pH 6.5 or pH 5.0) to the concentration of 0.5 mg/mL, respectively. Then, these samples were separately co-incubated with bovine serum albumin (BSA, 0.25 mg/mL) in a water bath shaker at 37 °C. After 2 h, the mixtures were centrifuged at 8000 rpm for 10 min. The supernatant (20 μL) of each sample and BCA assay medium (200 μL) were added into a 96-well plate, mixed and co-incubated at 37 °C for 30 min. Afterward, the absorbance at 562 nm was measured with a microplate reader (Thermo Scientific, USA). The concentration of residual BSA in each sample was calculated according to a prepared calibration curve with BSA standard samples.

### Hemolysis assay

The fresh whole blood (2 mL) of SD mice was supplied by the Laboratory Animal Center (Wuhan University), and centrifuged at 750 rpm for 10 min. The obtained red blood cells (RBC) were washed with saline until the supernatant was colourless. Then, RBC were resuspended in saline at 2% hematocrit. Afterward, the RBC suspension (0.8 mL) was separately incubated with 0.2 mL of LPNPs at a series of concentrations, saline (negative control), and ultrapure water (positive control) at 37 °C for 2 h. Subsequently, the mixtures were centrifuged at 1500 rpm for 15 min. The supernatant (200 μL) of each sample was separately added into a 96-well plate and the absorbance at 540 nm was measured using a microplate reader. The hemolysis rate was calculated according to the following formula:

Hemolysis (%) = (A_sample_ - A_negative_) / (A_positive_ - A_negative_) × 100

A_sample_ represents the absorbance of the test samples. A_positive_ and A_negative_ represent the absorbance of the positive control and negative control, respectively.

### *In vitro* release of DOX

The solutions of DOX-LPNPs (1 mL) were separately encapsulated in a dialysis tube (MWCO 3500 Da) and immerged into 10 mL of PBS (0.01M) at pH 7.4, pH 6.5 or pH 5.0. Then, these samples were incubated in a water bath shaker at 37 °C. At the designed time intervals, 3 mL of the external buffer of each sample was withdrawn and replaced with corresponding fresh PBS. The fluorescence intensity of released DOX in the external buffer was measured using RF-5301 PC spectrofluorophotometer (λ_ex_ = 488 nm, and λ_em_ = 590 nm) and the concentration of DOX was calculated according to the pre-established calibration curve.

### Cell line and cell culture

DOX-resistant MCF-7/ADR cells were supplied by Bena Culture Collection (Beijing, China) and incubated in RPMI 1640 medium (Hyclone^®^) containing 1 μg/mL DOX, 10% fetal bovine serum (Gibco^®^), and 1% penicillin-streptomycin solution (Hyclone^®^). Cells were kept in a humidified incubator containing 5% CO_2_ at 37 °C.

### Cellular uptake and mechanism analysis

Flow cytometry (CyAN-ADP, Beckman) was used to quantitatively evaluate the cellular uptake of DOX. Briefly, MCF-7/ADR cells were seeded in six-well plates (2×10^5^ cells per well) and incubated for 24 h to reach a confluence of 80%. Afterward, free DOX and DOX-LPNPs were separately added into the medium at a DOX concentration of 10 μg/mL and then incubated for another 24 h at pH 7.4 or pH 6.5. After washing with PBS three times, the cells were harvested and analyzed with flow cytometry.

To study the cellular uptake mechanism, MCF-7/ADR cells were seeded in six-well plates (2×10^5^ cells per well) and incubated for 24 h. Then, cells were incubated at 4 °C for 1 h, or separately pretreated with or without cytochalasin D (Cyt. D, 5 mM), methyl-β-cyclodextrin (MBCD, 5 mM), sucrose (0.45 M) at 37 °C for 1 h. Afterward, the medium was removed and replaced with fresh medium containing DOX-PLGA/CPT/PD at a DOX concentration of 10 μg/mL and incubated for another 24 h at pH 7.4 or pH 6.5. After washing with PBS three times, the cells were harvested and analyzed with flow cytometry.

### Intracellular distribution study

MCF-7/ADR cells were cultured in confocal laser dishes (1×10^5^ cells per dish) for 24 h. Free DOX and DOX-LPNPs were separately added into the medium at a DOX concentration of 10 μg/mL and then incubated for another 4 h or 12 h at pH 7.4 or pH 6.5. Subsequently, the cells were washed with PBS three times and then stained with 250 nM Lysotracker Green for 30 min at 37 °C in the dark. After washing with PBS three times, the cells were immediately observed using confocal laser scanning microscopy (CLSM, PerkinElmer Ultra VIEW VoX). The green fluorescence of Lysotracker Green and red fluorescence of DOX were separately detected using 488/525 nm and 488/600 nm excitation/emission filters. The Pearson's correlation coefficient of green fluorescence and red fluorescence was calculated using Imaging J software (National Institute of Health, USA).

Next, we investigated the mitochondria co-localization of DOX formulations. After treatment with DOX formulation (10 μg/mL) for 24 h at pH 7.4 or pH 6.5, MCF-7/ADR cells were stained with 100 nM Mitotracker Green for 15 min at 37 °C in the dark and then washed with PBS three times. Afterward, the cells were immediately observed using CLSM. The green fluorescence of Mitotracker Green and red fluorescence of DOX were separately detected using 488/525 nm and 488/600 nm excitation/emission filters.

### Measurement of DOX contents in the whole cells, nuclei, and mitochondria

MCF-7/ADR cells were incubated in cell-culture dishes with diameter of 100 mm to reach a confluence of 80%. Then, free DOX and DOX-LPNPs were separately added into the medium (10 mL). The final concentration of DOX was 10 μg/mL. The cells were incubated for another 24 h at pH 7.4 or pH 6.5. Afterward, the cells were harvested and washed three times with PBS. After resuspension with 1mL PBS, cells were equally divided into two parts. One part was used for the measurement of DOX content in the whole cells, and another part was used for the isolation of mitochondria. The first part of cells were lysed with lysis buffer (200 μL) at 4 °C, followed by centrifuging at 10000 rpm for 10 min. Then, the supernatant of each sample was collected, respectively. 20 μL of the supernatant was used to measure the content of protein, and 150 μL supernatant was added into the mixture of chloroform and isopropanol (300 μL, 4 : 1, v/v) to extract DOX. The organic layer was collected and dried at 60 °C overnight. The residues were separately dissolved in DMF (500 μL) and the DOX content of each sample was detected using RF-5301 PC spectrofluorophotometer. A mitochondria isolation kit was used to isolate mitochondria from the second part of cells according to the instruction. The obtained mitochondria were then lysed with mitochondrial lysate (200 μL). Afterward, the contents of protein and DOX in mitochondria were separately detected similar to the method described above.

The nuclei of the DOX-formulation (10 μg/mL) treated MCF-7/ADR cells (5×10^6^) were isolated according to the instruction of a nuclei extraction kit. The obtained nuclei were subsequently lysed, and the contents of protein and DOX were measured in a similar way described above.

### *In vitro* cytotoxicity assay

MCF-7/ADR cells were seeded in 96-well plates (1×10^4^ cells per well) and incubated at 37 °C for 24 h. Afterward, media containing gradient concentrations of free DOX or DOX-LPNPs were separately added into each well at pH 7.4 or pH 6.5. After incubation for another 48 h, the medium was removed and replaced with MTT-containing medium (100 μL, 0.5 mg/mL). 4 h later, the medium was removed and DMSO (150 μL) were added to each well to dissolve the produced formazan crystals. The absorbance at 570 nm was measured using a microplate reader. The cell viabilities (%) were calculated according to the following equation: Cell viability (%) = [(A_sample_-A_0_)/(A_control_-A_0_)] × 100. A_sample_ and A_control_ represent the absorbance values of cells treated with and without DOX formulations, respectively. A_0_ is the absorbance of the untreated cells without addition of MTT. The cytotoxicity of LPNPs without loading DOX against MCF-7/ADR or COS 7 cells was measured in a similar way as that of DOX-LPNPs.

### Mitochondrial membrane potentials (∆Ψ_m_)

JC-1 was used to investigate the mitochondrial membrane potentials (∆Ψ_m_). Briefly, MCF-7/ADR cells were cultured in confocal laser dishes (1×10^5^ cells per dish) for 24 h. Afterward, free DOX and DOX-LPNPs were separately added into the medium at pH 7.4 or pH 6.5. The concentration of DOX was 20 μg/mL. After treatment for another 24 h, the cells were washed three times with PBS and stained with 10 μg/mL JC-1 for 15 min at 37 °C in the dark. Subsequently, the cells were rinsed with PBS three times and immediately observed by CLSM. The green fluorescence of J-monomer and the red fluorescence of J-aggregates were detected with 488/525 nm and 561/600 nm excitation/emission filters, respectively.

### Mitochondrial bioenergetics analysis

Seahorse XF24 extracellular flux analyzer was used to detect oxygen consumption rate (OCR) of cells. MCF-7/ADR cells were seeded in a XF24-well cell plate (2.5×10^4^ cells per well) and incubated in 5% CO_2_ atmosphere at 37 °C overnight. Afterward, the medium containing free DOX or DOX-LPNPs at DOX concentration of 20 μg/mL was separately added into each well at pH 6.5. After incubation for another 24 h, all but 50 μL of the medium was removed and cells were rinsed twice with 1 mL XF stress assay medium. Then, 450 µL assay medium was added to each well, and the cell plate was placed in a non-CO_2_ incubator at 37 °C for 1 h before assay. The OCR of treated or untreated MCF-7/ADR cells was measured before and after the successive addition of oligomycin (10 µM), FCCP (10 μM), and rotenone/antimycin-A (5 µM) according to the supplier's construction.

### Western Blot analysis

MCF-7/ADR cells were incubated in six-well plates (2×10^5^ cells per well) for 24 h. Then, free DOX and DOX-LPNPs were separately added into each well at pH 6.5. The concentration of DOX was 20 μg/mL. After incubation for another 24 h, the cells were washed three times with PBS and lysed with lysis buffer at 4 °C for 10 min. The lysates were subjected to SDS-PAGE and then transferred onto PVDF membranes (Millipore). After being blocked in 5% skim milk for 1 h, the membranes were separately incubated with rabbit antibodies against cytochrome c (cyt. c), caspase-9, caspase-3, Bcl-2, Bax and β-actin at 4 °C overnight, and then treated with secondary antibody HRP-goat anti-rabbit (1:10000) for 30 min at room temperature. Subsequently, the immunoreactive bands were visualized by enhanced chemiluminescence (ECL; Pierce). β-Actin was detected as a protein loading control.

### Animals and tumor model

All animal experiments were performed according to the regulations for laboratory animals established by the Wuhan University Center for Animal Experiment/A3-Lab with the approval of the Wuhan University of China Animal Care and Use Committee. Female KM mice (6 weeks old) for pharmacokinetics study and female athymic BALB/c-nude mice (4-5 weeks old, 16 ± 2 g) for the construction of a tumor model were supplied by Beijing HFK Bioscience Co. Ltd (Beijing, China). Briefly, MCF-7/ADR cells (5×10^6^ cells) with 50% matrigel matrix were injected subcutaneously into the right back of each mouse to establish a tumor model. A caliper was used to measure the length (L) and width (W) of the tumor. Tumor volume (V, mm^3^) was calculated according to the formula: V = L×W^2^/2.

### Pharmacokinetics study

Free DOX and DOX-LPNPs were intravenously injected into KM mice at a dose of 5 mg DOX per kg body weight, respectively (n = 3). At the predetermined time point (1, 2, 3, 4, 6, 9, 12, 24 h), 50 μL of blood samples were separately collected from the tail vein, and added into 200 μL PBS containing heparin sodium. Then, the samples were centrifuged at 3000 rpm for 5 min. 50 μL HCl (5 M) was added into the supernatants, followed by incubation at 50 °C for 2 h. Afterward, 50 μL NaOH (5 M) was added into each sample and DOX was extracted with 300 μL of chloroform and isopropanol (4 : 1, v/v). The organic layer was collected and dried at 60 °C in the dark overnight. The residue was dissolved in 150 μL of acetonitrile and water (55 : 45, v/v) and filtered through a syringe filter (pore size = 220 nm). Finally, the content of DOX in each sample was detected using HPLC analysis (SHIMADZU, LC-15C, Kyoto, Japan) equipped with a reverse-phase Angel C18 column (250×4.6 mm^2^, 5 mm) and a fluorescence detector. The excitation/emission wavelength was 488/590 nm. Acetonitrile/water (55 : 45, v/v) was used as the mobile phase at a flow rate of 1 mL/min. To establish the calibration curve, DOX at a certain concentration was added into the whole blood of untreated mice and then extracted in a similar way as describe above. Finally, the pharmacokinetics parameters of free DOX and DOX-LPNPs were calculated by DAS 3.0 software.

### Biodistribution of DOX *in vivo*

When the tumors of BALB/c-nude mice reached approximate 100 mm^3^, mice were randomly divided into 5 groups (3 mice per group). Then, free DOX and DOX-LPNPs were intravenously injected at a dose of 5 mg DOX per kg body weight, respectively. After 24 h, the mice were sacrificed, and the tumors as well as major organs including heart, liver, spleen, lung, and kidney were harvested and imaged using living image IVIS^®^ spectrum (PerkinElmer).

For the further quantitative analysis of DOX biodistribution, the collected tumors and organs were separately weighed and then grinded in KH_2_PO_4_ solution (1.5 mL, 0.02 mM) using a tissue pulverizer (IKA, T 25 digital S 25, Germany). Then the tissue homogenate (200 μL) was acidolysis with 50 μL HCl (5 M) at 60 °C. After 2 h, 50 μL NaOH (5 M) was added into each sample and DOX was extracted and measured in a similar way as that of pharmacokinetics study. DOX at a certain concentration was added into the tissues of untreated mice before homogenization to establish the calibration curve.

### Measurement of DOX contents in cancer cells and their mitochondria *in vivo*

MCF-7/ADR-bearing mice were divided into 5 groups (3 mice per group) randomly after the tumor volume reached approximate 200 mm^3^. Afterward, free DOX and DOX-LPNPs were intravenously injected at a dose of 5 mg DOX per kg body weight, respectively. 24 h later, the mice were sacrificed, and the tumors were collected and washed twice with Hank's buffer. Then tumors were cut into pieces (1 mm^3^), followed by treatment with the mixture of collagenase I (1.2 mg/mL) and collagenase IV (2 mg/mL) in Hank's buffer at 37 °C for 4 h. After being filtered by cell filtration sieves (40 mesh), the cancer cells were extracted using differential adhesion method 41. The obtained cancer cells were equally divided into two parts, and the separation of DOX from the whole cancer cells and their mitochondria was same as that described above. The contents of DOX were measured using HPLC analysis.

### *In vivo* therapeutic study

For the *in vivo* antitumor study, MCF-7/ADR-bearing mice were randomly divided into 6 groups (6 mice per group) after the tumors reached approximate 100 mm^3^. Then, PBS, free DOX, DOX-PLGA/CPT, DOX-PLGA/CPO, DOX-PLGA/CPT/PS, and DOX-PLGA/CPT/PD were intravenously injected every three days at a dose of 5 mg DOX per kg body weight, respectively. The tumor volumes and body weights of mice were monitored every other day. After treatment for 18 days, the mice were sacrificed and the tumor tissues as well as the main organs were collected for histological and immunohistochemical analyses. Tumor inhibitory rate (TIR) was calculated according to the following formula: TIR (%) = [1- (V_tf_ - V_ti_)/(V_pf_ - V_pi_)] × 100, where V_tf_ and V_ti_ separately represent the final and initial tumor volume of the treated group, while V_pf_ and V_pi_ separately represent the final and initial tumor volume of the PBS group.

### Statistical analysis

Statistical analyses were performed using Student's *t*-test. For all analyses, *p < 0.05, **p < 0.01, and ***p < 0.001 were considered to be statistically significant. Data were expressed as means ± standard deviation (SD).

## Abbreviations

EPR effect: enhanced permeability and retention effect
MDR: multidrug resistance
TME: tumor microenvironment
DOX: doxorubicin
TOPOII: topoisomerase II
P-gp: P-glycoprotein
LPNPs: lipid-polymer hybrid nanoparticles
TPP: triphenylphosphonium
CPT: C_18_-PEG_2000_-TPP
CPO: C_18_-PEG_2000_-OH
PEI: polyethylenimine
DMMA: 2,3-dimethylmaleic anhydride
SA: succinic anhydride
PD: PEI-DMMA
PS: PEI-SA
DLC: drug loading content
PDI: polydispersity index
TACMAA: tumor-acidity-cleavable maleic acid amide
RES: reticuloendothelial system
BSA: bovine serum albumin
RBC: red blood cells
Cyt. D: Cytochalasin D
MBCD: methyl-β-cyclodextrin
FCM: flow cytometry
CLSM: confocal laser scanning microscopy
OCR: oxygen consumption rate
Cyt. C: cytochrome c
TIR: tumor inhibitory rate

## References

[1] Holohan C, Schaeybroeck SV, Longley DB, Johnston PG (2013). Cancer drug resistance: an evolving paradigm. Nat Rev Cancer.

[2] Dagogo-Jack I, Shaw AT (2017). Tumour heterogeneity and resistance to cancer therapies. Nat Rev Clin Oncol.

[3] Huang QQ, Cai TG, Bai L, Huang YH, Li QW, Wang Q (2019). State of the art of overcoming efflux transporter mediated multidrug resistance of breast cancer. Transl Cancer Res.

[4] Szakacs G, Paterson JK, Ludwig JA, Booth-Genthe C, Gottesman MM (2006). Targeting multidrug resistance in cancer. Nat Rev Drug Discovery.

[5] Pan ST, Li ZL, He ZX, Qiu JX, Zhou SF (2016). Molecular mechanisms for tumour resistance to chemotherapy. Clin Exp Pharmacol Physiol.

[6] Higgins CF (2007). Multiple molecular mechamisms for multidrug resistance transporters. Nature.

[7] Gao ZB, Zhang LN, Sun YJ (2012). Nanotechnology applied to overcome tumor drug resistance. J Controlled Release.

[8] Li R, Xie Y (2017). Nanodrug delivery systems for targeting the endogenous tumor microenvironment and simultaneously overcoming multidrug resistance properties. J Controlled Release.

[9] Zhou G, Latchoumanin O, Hebbard L, Duan W, Liddle C, George J (2018). Aptamers as targeting ligands and therapeutic molecules for overcoming drug resistance in cancers. Adv Drug Delivery Rev.

[10] Zhou L, Wang H, Li YP (2018). Stimuli-responsive nanomedicines for overcoming cancer multidrug resistance. Theranostics.

[11] Li HJ, Du JZ, Du XJ, Xu CF, Sun CY, Wang HX (2016). Stimuli-responsive clustered nanoparticles for improved tumor penetration and therapeutic efficacy. Proc Natl Acad Sci USA.

[12] Wei X, Wang Y, Xiong X, Guo X, Zhang L, Zhang XB (2016). Codelivery of a pi-pi stacked dual anticancer drug combination with nanocarriers for overcoming multidrug resistance and tumor metastasis. Adv Funct Mater.

[13] Chen MM, Song FF, Liu Y, Tian J, Liu C, Li RY (2019). A dual pH-sensitive liposomal system with charge-reversal and NO generation for overcoming multidrug resistance in cancer. Nanoscale.

[14] Ye MZ, Han YX, Tang JB, Piao Y, Liu XR, Zhou ZX (2017). A tumor-specific cascade amplification drug release nanoparticle for overcoming multidrug resistance in cancers. Adv Mater.

[15] Wang Q, Zhang XY, Liao HZ, Sun Y, Ding L, Teng YW (2018). Multifunctional shell core nanoparticles for treatment of multidrug resistance hepatocellular carcinoma. Adv Funct Mater.

[16] Zhang YJ, Leonard M, Shu Y, Yang YG, Shu D, Guo PX (2017). Overcoming tamoxifen resistance of human breast cancer by targeted gene silencing using multifunctional pRNA nanoparticles. ACS Nano.

[17] Marrache S, Pathak RK, Dhar S (2014). Detouring of cisplatin to access mitochondrial genome for overcoming resistance. Proc Natl Acad Sci USA.

[18] Guo X, Wei X, Jing YT, Zhou SB (2015). Size changeable nanocarriers with nuclear targeting for effectively overcoming multidrug resistance in cancer therapy. Adv Mater.

[19] Lei XP, Chen MF, Li XB, Huang MH, Nie QL, Ma N (2018). A vascular disrupting agent overcomes tumor multidrug resistance by skewing macrophage polarity toward the M1 phenotype. Cancer Lett.

[20] Li FQ, Mei H, Gao Y, Xie XD, Nie HF, Li T (2017). Co-delivery of oxygen and erlotinib by aptamer-modified liposomal complexes to reverse hypoxia-induced drug resistance in lung cancer. Biomaterials.

[21] Shen S, Li HJ, Chen KG, Wang YC, Yang XZ, Lian ZX (2017). Spatial targeting of tumor-associated macrophages and tumor cells with a pH-sensitive cluster nanocarrier for cancer chemoimmunotherapy. Nano Lett.

[22] Chamberlain GR, Tulumello DV, Kelley SO (2013). Targeted delivery of doxorubicin to mitochondria. ACS Chem Biol.

[23] Dartier J, Lemaitre E, Chourpa I, Goupille C, Servais S, Chevalier S (2017). ATP-dependent activity and mitochondrial localization of drug efflux pumps in doxorubicin-resistant breast cancer cells. Biochim Biophys Acta.

[24] Ma P, Mumper RJ, Anthracycline nano-delivery systems to overcome multiple drug resistance (2013). a comprehensive review. Nano Today.

[25] Jung HS, Han J, Lee JH, Lee JH, Choi JM, Kweon HS (2015). Enhanced NIR radiation-triggered hyperthermia by mitochondrial targeting. J Am Chem Soc.

[26] Guo RR, Peng HB, Tian Y, Shen S, Yang WL (2016). Mitochondria-targeting magnetic composite nanoparticles for enhanced phototherapy of cancer. Small.

[27] Luo SL, Tan X, Fang ST, Wang Y, Liu T, Wang X (2016). Mitochondria-targeted small-molecule fluorophores for dual modal cancer phototherapy. Adv Funct Mater.

[28] Buondonno I, Gazzano E, Jean SR, Audrito V, Kopecka J, Fanelli M (2016). Mitochondria targeted doxorubicin: a new therapeutic strategy against doxorubicin-resistant osteosarcoma. Mol Cancer Ther.

[29] Yu PC, Yu HJ, Guo CY, Cui ZR, Chen XZ, Yin Q (2015). Reversal of doxorubicin resistance in breast cancer by mitochondria-targeted pH-responsive micelles. Acta Biomater.

[30] Zhang Y, Zhang CJ, Chen J, Liu L, Hu MY, Li J (2017). Trackable mitochondria-targeting nanomicellar loaded with doxorubicin for overcoming drug resistance. ACS Appl Mater Interfaces.

[31] Li WQ, Wang ZG, Hao SJ, He HZ, Wan Y, Zhu CD (2017). Mitochondria-targeting polydopamine nanoparticles to deliver doxorubicin for overcoming drug resistance. ACS Appl Mater Interfaces.

[32] Marrache S, Dhar S (2012). Engineering of blended nanoparticle platform for delivery of mitochondria-acting therapeutics. Proc Natl Acad Sci USA.

[33] Cho DY, Cho H, Kwon K, Yu M, Lee E, Huh KM (2015). Triphenylphosphonium conjugated poly(epsilon-caprolactone)-based self-assembled nanostructures as nanosized drugs and drug delivery carriers for mitochondria-targeting synergistic anticancer drug delivery. Adv Funct Mater.

[34] Han M, Vakili MR, Abyaneh HS, Molavi O, Lai R, Lavasanifar A (2014). Mitochondrial delivery of doxorubicin via triphenylphosphine modification for overcoming drug resistance in MDA-MB-435/DOX cells. Mol Pharm.

[35] Liu YQ, Zhang XJ, Zhou MJ, Nan XY, Chen XF, Zhang XH (2017). Mitochondrial-targeting lonidamine-doxorubicin nanoparticles for synergistic chemotherapy to conquer drug resistance. ACS Appl Mater Interfaces.

[36] Liu HN, Guo NN, Guo WW, Huang-Fu MY, Vakili MR, Chen JJ (2018). Delivery of mitochondriotropic doxorubicin derivatives using self-assembling hyaluronic acid nanocarriers in doxorubicin-resistant breast cancer. Acta Pharmacol Sin.

[37] Liu HN, Guo NN, Wang TT, Guo WW, Lin MT, Huang-Fu MY (2018). Mitochondrial targeted doxorubicin-triphenylphosphonium delivered by hyaluronic acid modified and pH responsive nanocarriers to breast tumor: *in vitro* and *in vivo* studies. Mol Pharm.

[38] Zhou W, Yu H, Zhang LJ, Wu B, Wang CX, Wang Q (2017). Redox-triggered activation of nanocarriers for mitochondria-targeting cancer chemotherapy. Nanoscale.

[39] Du JZ, Li HJ, Wang J (2018). Tumor-acidity-cleavable maleic acid amide (TACMAA): a powerful tool for designing smart nanoparticles to overcome delivery barriers in cancer nanomedicine. Acc Chem Res.

[40] Jiang W, Wang JL, Yang JB, He ZW, Hou ZH, Luo YL (2018). Acidity-triggered TAT-presenting nanocarriers augment tumor retention and nuclear translocation of drugs. Nano Res.

[41] Biernaskie JA, McKenzie IA, Toma JG, Miller FD (2006). Isolation of skin-derived precursors (SKPs) and differentiation and enrichment of their Schwann cell progeny. Nat Protoc.

