# Supplementary material for: Tumor acidity activated triphenylphosphonium-based mitochondrial targeting nanocarriers for overcoming drug resistance of cancer therapy
Source: Theranostics. 2019 Sep 21;9(23):7033–50. doi: 10.7150/thno.35748 (PMC6815947; doi:10.7150/thno.35748)
Supplement: Supplementary file 1 — Supplementary figures and tables. [file thnov09p7033s1.pdf]

**Supporting Information**

# Tumor acidity activated triphenylphosphonium-based mitochondrial targeting nanocarriers for overcoming drug resistance of cancer therapy

Hui Yu, Jia-Mi Li, Kai Deng, Wei Zhou, Cai-Xia Wang, Qian Wang, Kun-Heng Li, Hong-Yang Zhao, Shi-Wen Huang*

Key Laboratory of Biomedical Polymers of Ministry of Education, Department of Chemistry, Wuhan University, Wuhan 430072, China

***Correspondence: e-mail: swhuang@whu.edu.cn

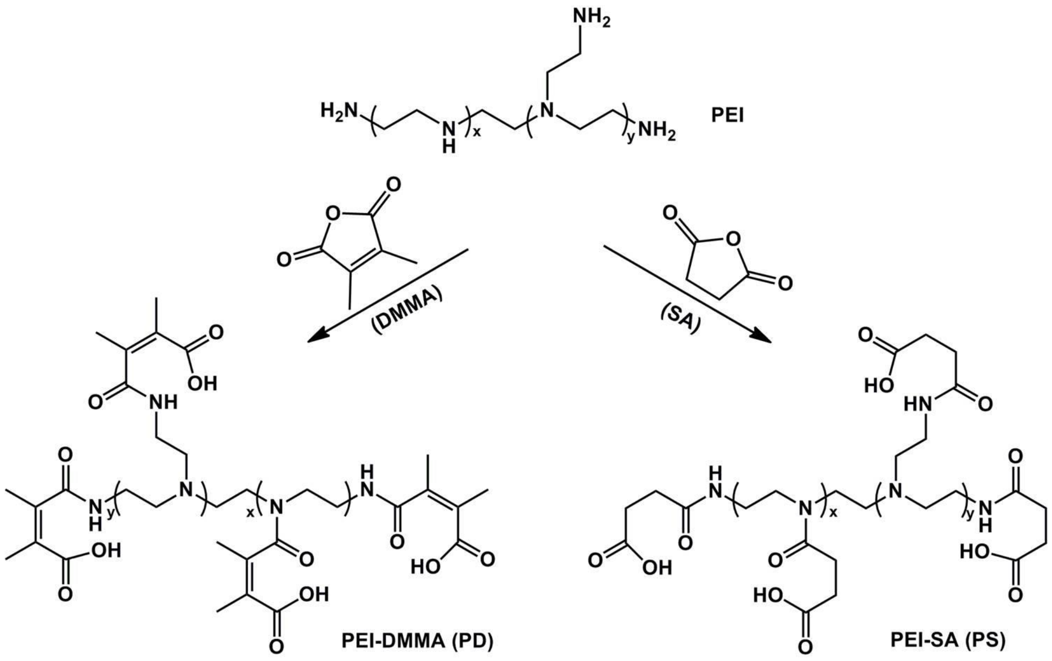


**Scheme S1**. Synthesis of PEI-DMMA (PD) and PEI-SA (PS).

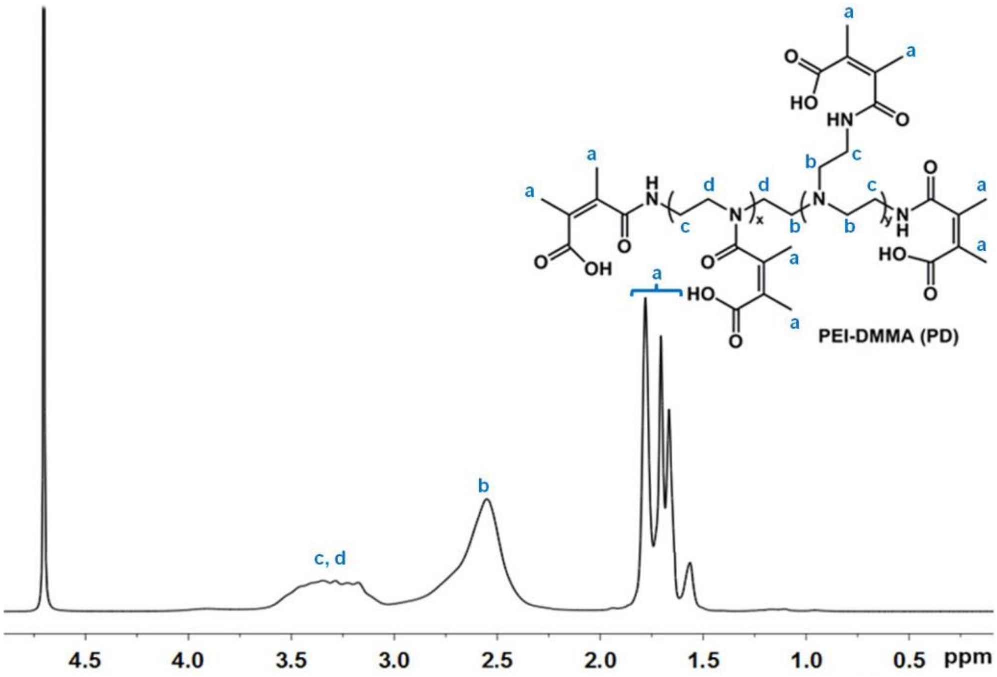


**Figure S1**. $^1$H NMR spectrum (400 MHz, $D_2O$) of PEI-DMMA (PD).

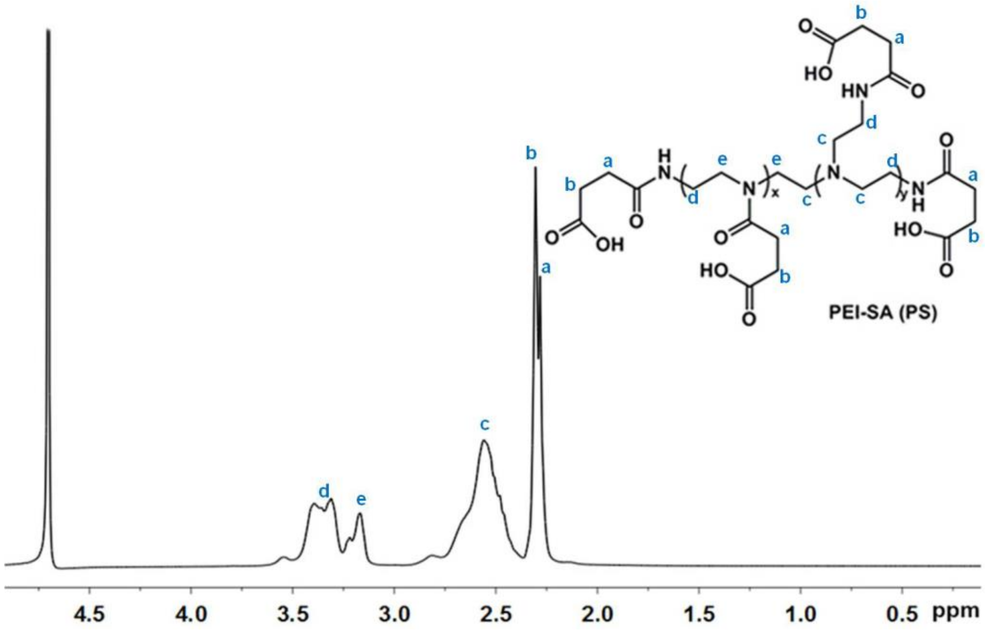


**Figure S2**. $^1$H NMR spectrum (400 MHz, $D_2O$) of PEI-SA (PS).

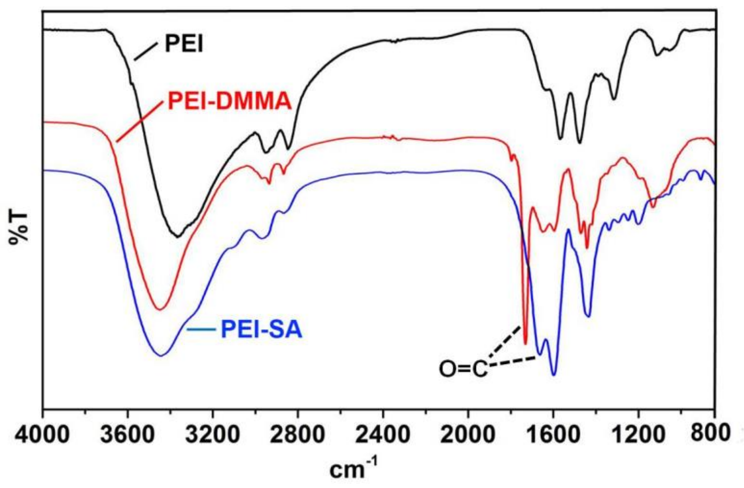


**Figure S3**. FT-IR spectra of PEI, PEI-DMMA (PD), and PEI-SA (PS).

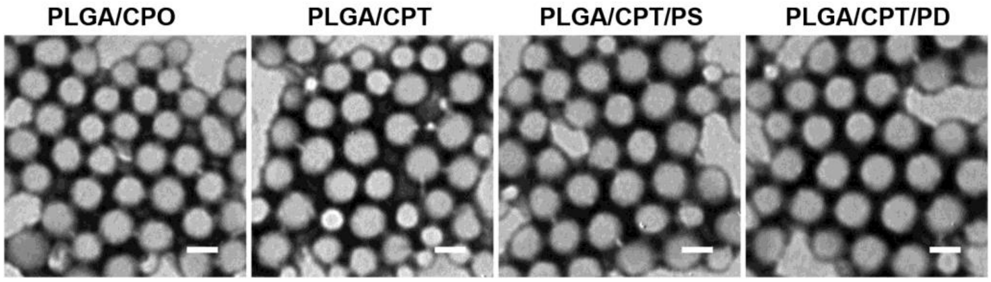


**Figure S4** TEM images of LPNPs, scale bar = 100 nm.

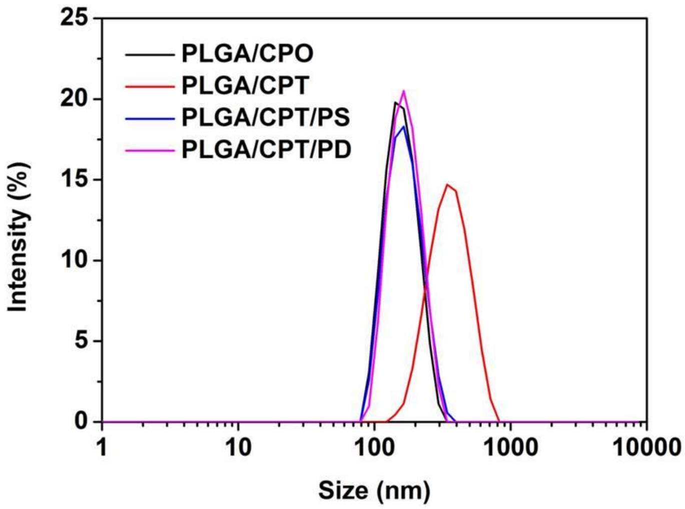


**Figure S5.** Hydrodynamic sizes of LPNPs in RPMI 1640 medium containing 10% fetal bovine serum (FBS).

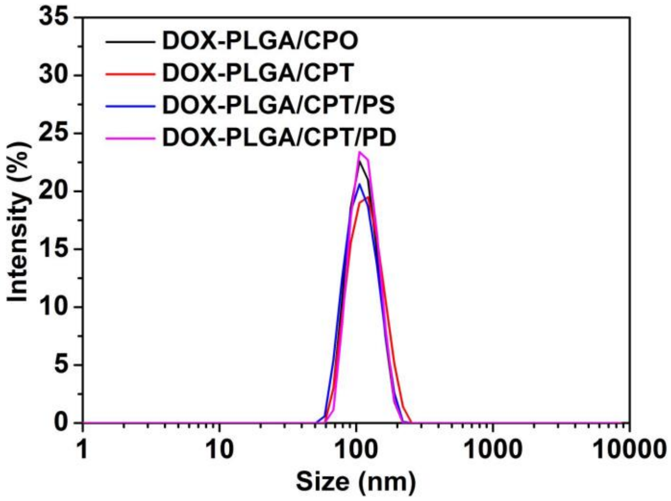


**Figure S6**. Hydrodynamic sizes of DOX loaded LPNPs in PBS.

**Table S1**. Characterization of LPNPs and DOX-LPNPs

| Samples | LPNPs in PBS | | | LPNPs in RPMI 1640 medium | | | DOX-LPNPs in PBS | | | |
|---|---|---|---|---|---|---|---|---|---|---|
| | Size (nm) | PDI | Zeta potential (mV) | Size (nm) | PDI | Zeta potential (mV) | Size (nm) | PDI | Zeta potential (mV) | DLC (%) |
| PLGA/CPO | 146.2 | 0.095 | -18.3 | 151.7 | 0.132 | -21.7 | 154.5 | 0.128 | -16.6 | 6.10 |
| PLGA/CPT | 149.3 | 0.233 | 19.6 | 319.2 | 0.381 | 7.45 | 164.1 | 0.240 | 21.8 | 5.82 |
| PLGA/CPT/PS | 159.7 | 0.169 | -31.2 | 164.9 | 0.152 | -35.1 | 161.8 | 0.179 | -32.8 | 5.39 |
| PLGA/CPT/PD | 156.6 | 0.124 | -24.2 | 166.6 | 0.154 | -29.6 | 162.7 | 0.162 | -18.9 | 5.66 |

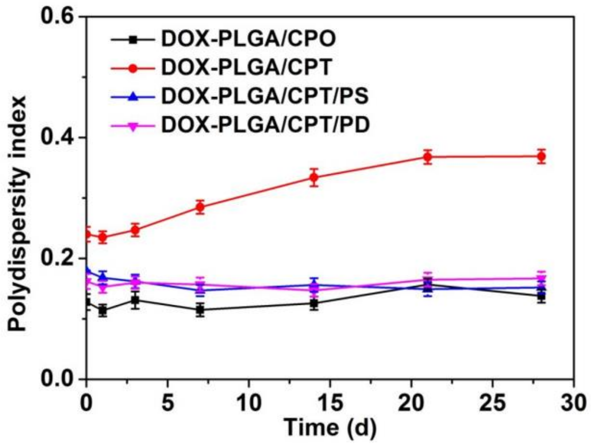


**Figure S7**. Polydispersity index (PDI) of DOX-LPNPs stored at 4 °C.

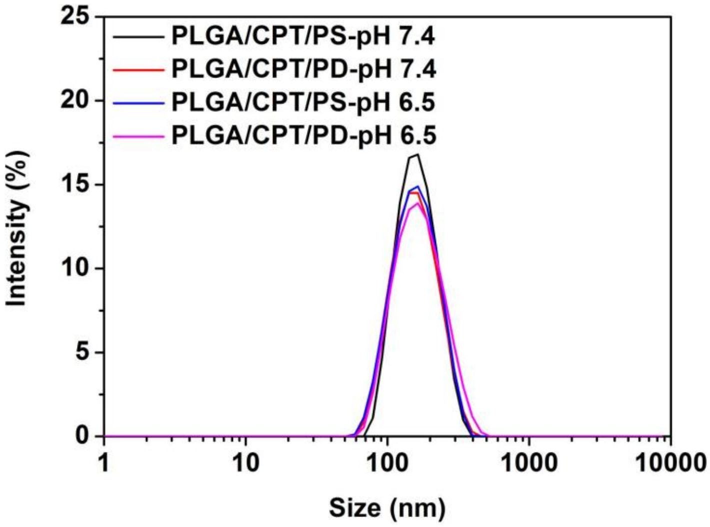


**Figure S8**. Sizes of PLGA/CPT/PS and PLGA/CPT/PD after separate treatment at pH 7.4 and pH 6.5 for 4 h.

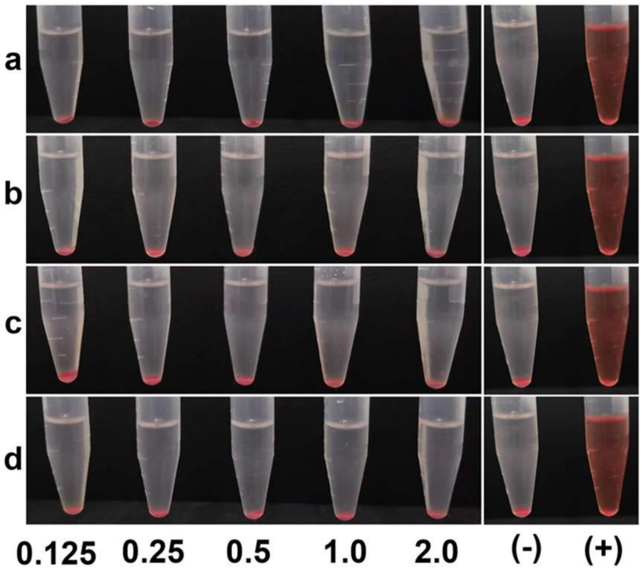


**Figure S9**. Photographs of hemolysis assay. a, PLGA/CPO; b, PLGA/CPT; c, PLGA/CPT/PS; d, PLGA/CPT/PD; (-), saline; (+), ultrapure water.

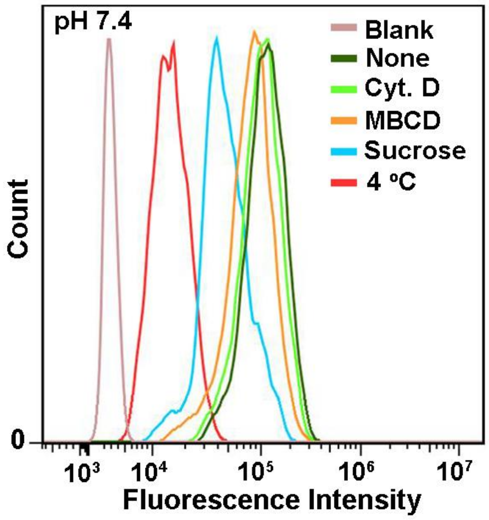


**Figure S10**. Cellular uptake mechanism analysis of DOX-PLGA/CPT/PD in MCF-7/ADR cells at pH 7.4.

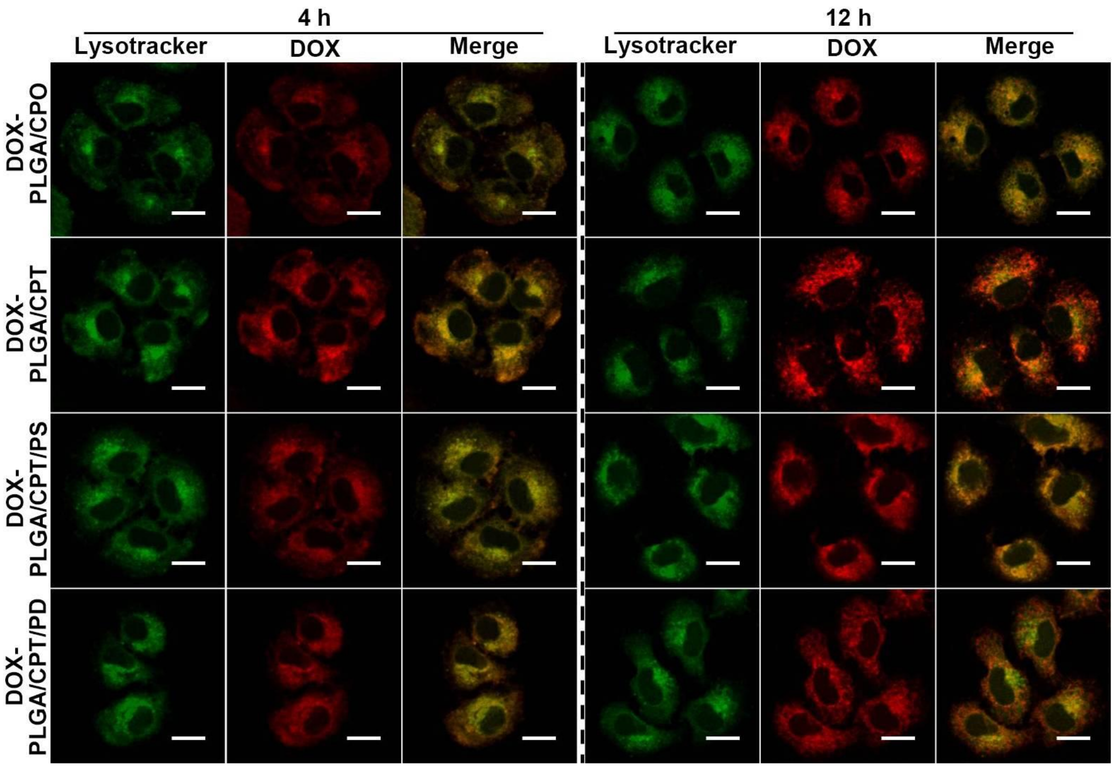


**Figure S11**. CLSM images of MCF-7/ADR cells after incubation with DOX-LPNPs for 4 h or 12 h at pH 7.4, scale bar = 20 μm.

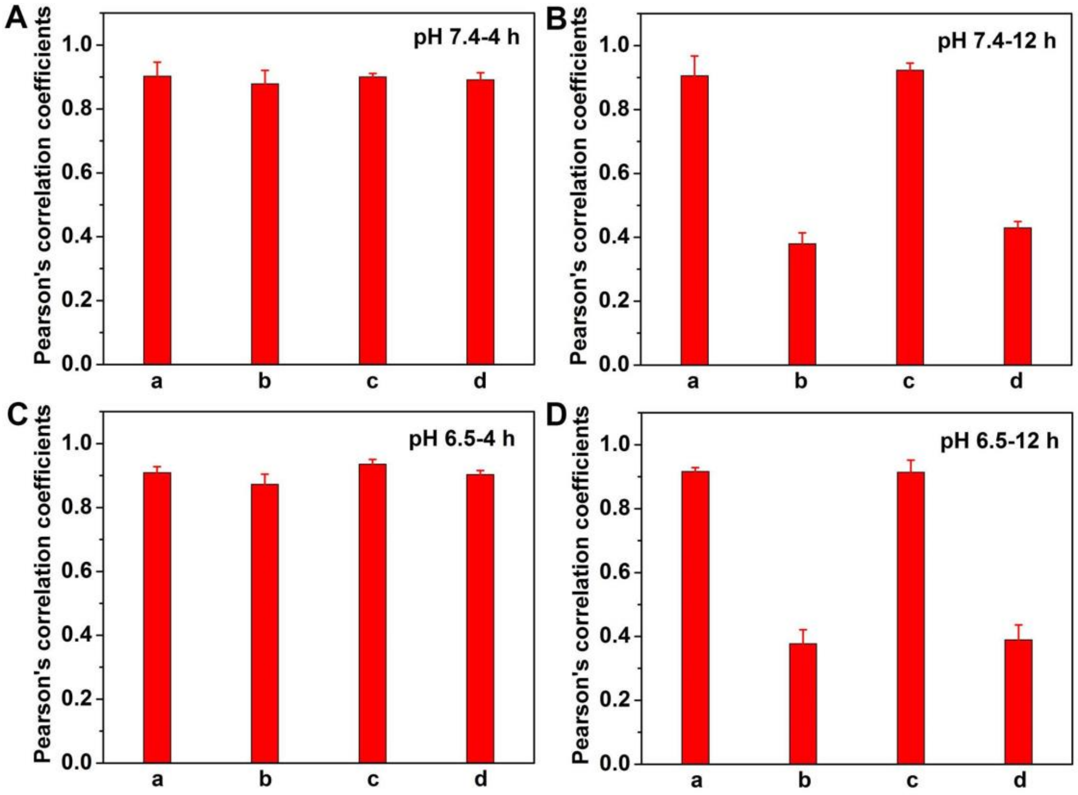


**Figure S12.** Pearson's correlation coefficients of green fluorescence from Lysotracker Green and red fluorescence from DOX. a, DOX-PLGA/CPO; b, DOX-PLGA/CPT; c, DOX-PLGA/CPT/PS; d, DOX-PLGA/CPT/PD.

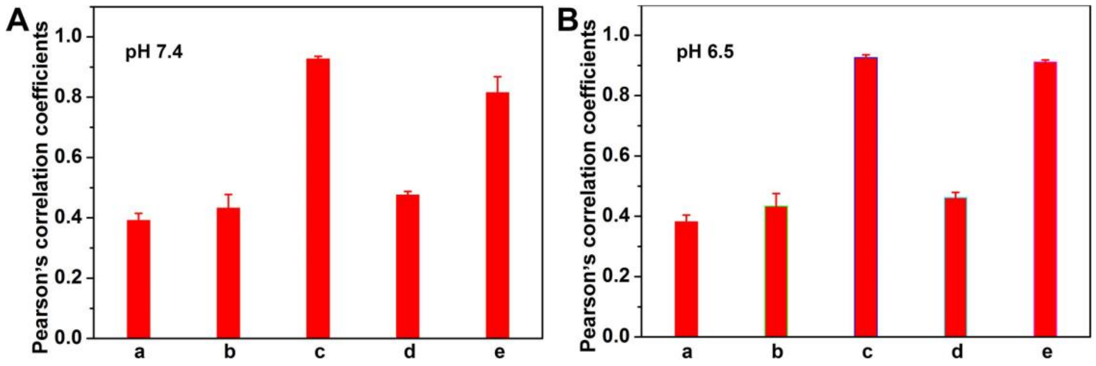


**Figure S13.** Pearson's correlation coefficients of green fluorescence from Mitotracker Green and red fluorescence from DOX. a, free DOX; b, DOX-PLGA/CPO; c, DOX-PLGA/CPT; d, DOX-PLGA/CPT/PS; e, DOX-PLGA/CPT/PD.

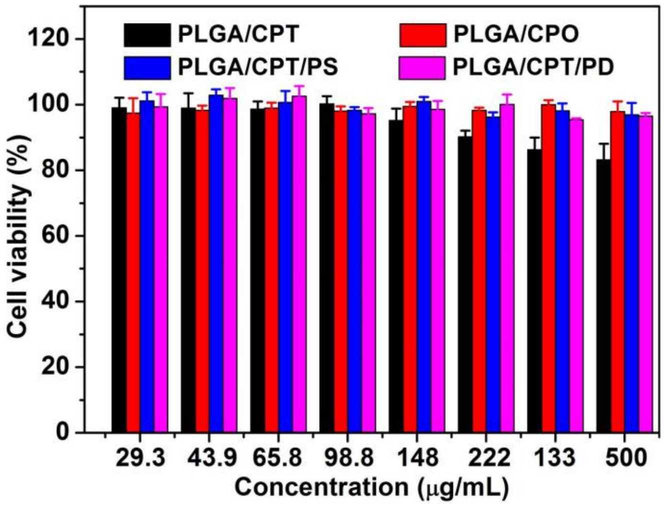


**Figure S14.** Cytotoxicity of LPNPs against COS 7 cells.

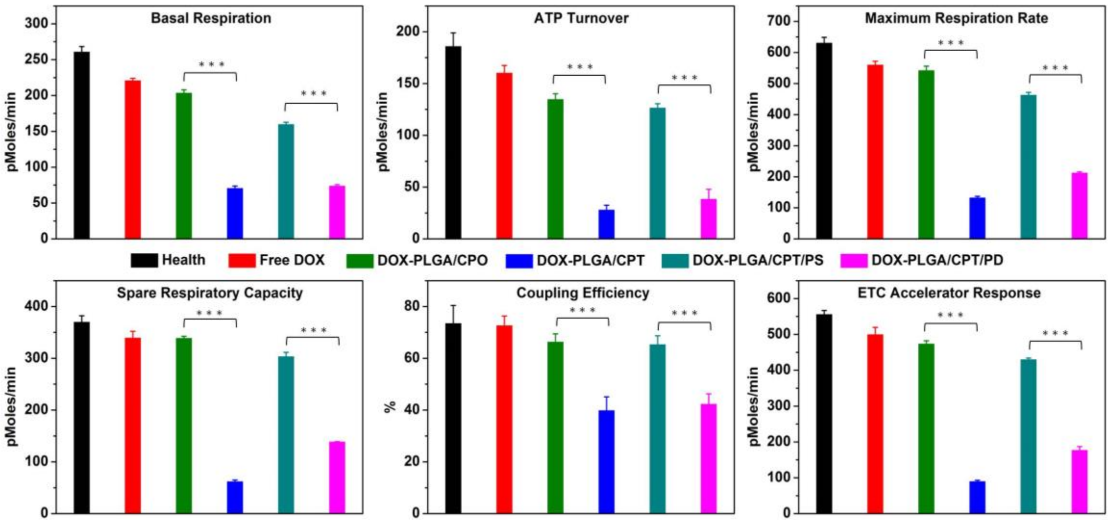


**Figure S15.** Comparison of basal respiration, ATP turnover, maximum respiration rate, spare respiratory capacity, coupling efficiency, and electron transport chain (ETC) accelerator response of MCF-7/ADR cells after treatment with or without DOX formulations at pH 6.5 for 24 h (mean ± S.D, n=3), ***P < 0.001.

**Table S2**. Pharmacokinetic parameters of free DOX and DOX-LPNPs after intravenous injection at a dose of 5 mg DOX per kg body weight (n = 3).

| Parameters | Free DOX | DOX-PLGA/CPT | DOX-PLGA/CPO | DOX-PLGA/CPT/PS | DOX-PLGA/CPT/PD |
|---|---|---|---|---|---|
| $t_{1/2Z}$ | 2.71 | 4.13 | 16.92 | 18.82 | 15.84 |
| $AUC_{0-\infty}$ | 25.09 | 35.90 | 112.79 | 122.35 | 109.60 |
| $MRT_{0-\infty}$ | 3.81 | 4.24 | 16.59 | 17.02 | 10.06 |
| $CL_Z$ | 223.69 | 199.25 | 55.80 | 44.33 | 65.88 |

Parameters and units: half-life ($t_{1/2Z}$, h); area under the curve ($AUC_{0-\infty}$, mg/L×h); mean residence time ($MRT_{0-\infty}$, h); total body clearance ($CL_Z$, L/h/kg).

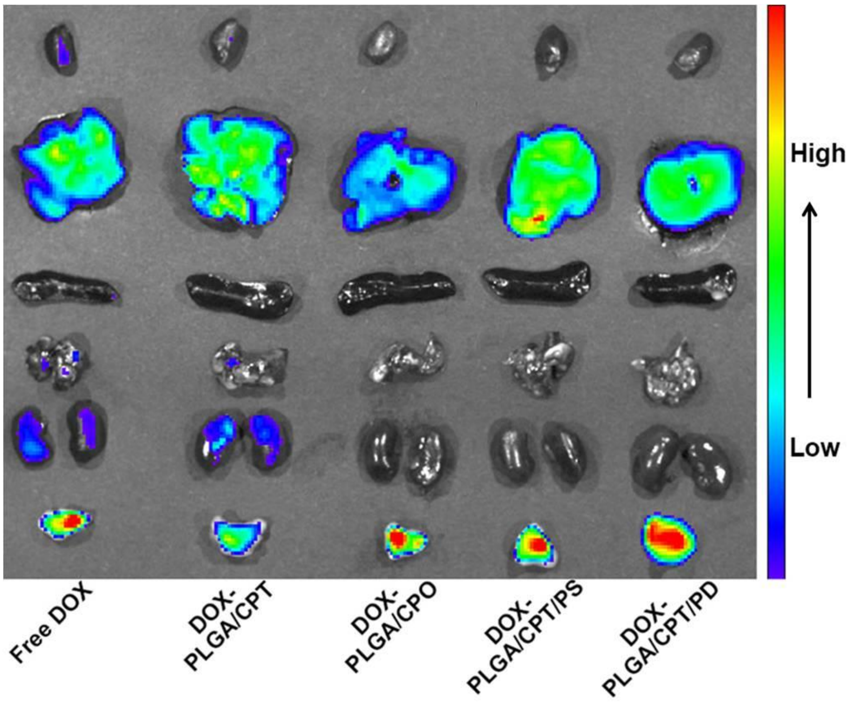


**Figure S16.** Fluorescence image of heart, liver, spleen, lung, kidney, and tumor of MCF-7/ADR tumor-bearing mice after intravenous injection for 24 h.

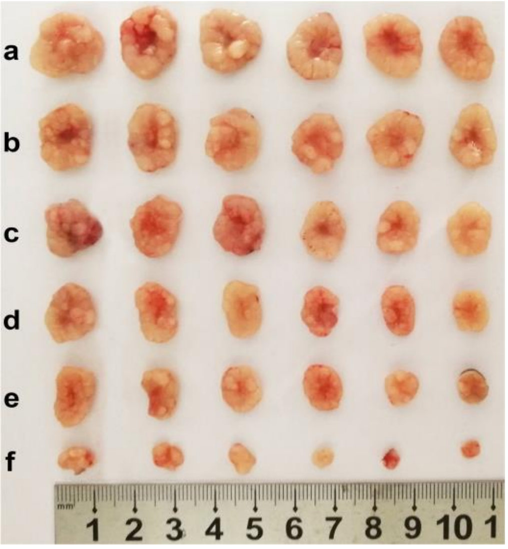


**Figure S17.** Tumor images on the 18th day after various treatments. a, PBS; b, free DOX; c, DOX-PLGA/CPT; d, DOX-PLGA/CPO; e, DOX-PLGA/CPT/PS; f, DOX-PLGA/CPT/PD.

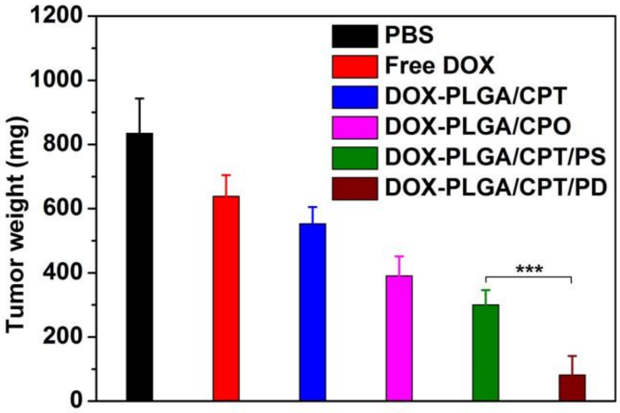


**Figure S18.** Weights of MCF-7/ADR tumors after various treatments for 18 days (mean ± S.D, n=3), ***$P < 0.001$.

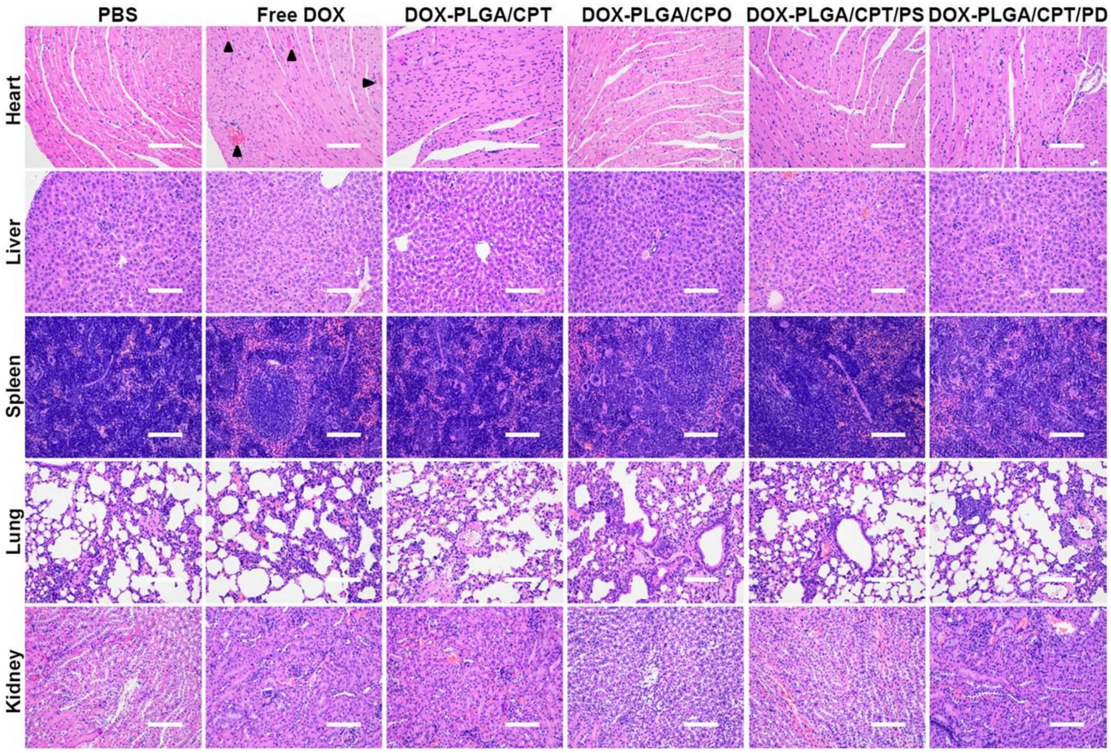


**Figure S19.** H&E staining analysis of mian organs after treatment with PBS, free DOX, DOX-PLGA/CPT, DOX-PLGA/CPO, DOX-PLGA/CPT/PS, and DOX-PLGA/CPT/PD for 18 days, scale bar = 100 μm.
